# Wnt9 directs zebrafish heart tube assembly via a combination of canonical and non-canonical pathway signaling

**DOI:** 10.1242/dev.201707

**Published:** 2023-09-25

**Authors:** Alessio Paolini, Dinara Sharipova, Tim Lange, Salim Abdelilah-Seyfried

**Affiliations:** Institute of Biochemistry and Biology, Potsdam University, D-14476 Potsdam, Germany

**Keywords:** Klf2, Wnt9, Cardiogenesis, Zebrafish, Morphogenesis

## Abstract

During zebrafish heart formation, cardiac progenitor cells converge at the embryonic midline where they form the cardiac cone. Subsequently, this structure transforms into a heart tube. Little is known about the molecular mechanisms that control these morphogenetic processes. Here, we use light-sheet microscopy and combine genetic, molecular biological and pharmacological tools to show that the paralogous genes *wnt9a*/*b* are required for the assembly of the nascent heart tube. In *wnt9a*/*b* double mutants, cardiomyocyte progenitor cells are delayed in their convergence towards the embryonic midline, the formation of the heart cone is impaired and the transformation into an elongated heart tube fails. The same cardiac phenotype occurs when both canonical and non-canonical Wnt signaling pathways are simultaneously blocked by pharmacological inhibition. This demonstrates that Wnt9a/b and canonical and non-canonical Wnt signaling regulate the migration of cardiomyocyte progenitor cells and control the formation of the cardiac tube. This can be partly attributed to their regulation of the timing of cardiac progenitor cell differentiation. Our study demonstrates how these morphogens activate a combination of downstream pathways to direct cardiac morphogenesis.

## Teaser

**Summary:** The role of *wnt9a*/*b* in assembly of myocardial progenitor cells at the embryonic midline to form the heart cone and then initiate leftward-directed heart tube elongation.

## INTRODUCTION

**Fig. 1.**
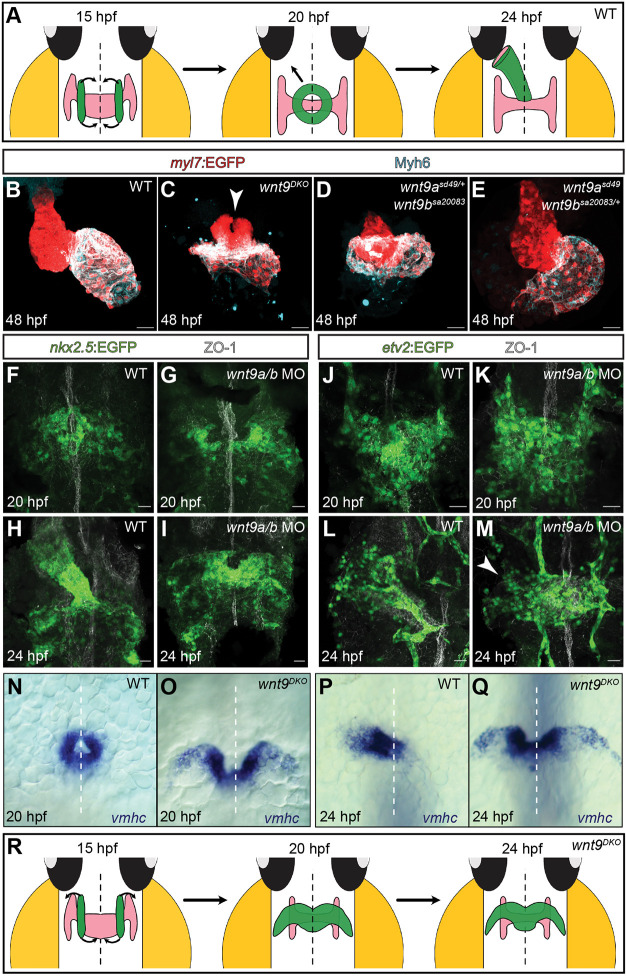
**The *wnt9a*/*b* paralogous genes are essential for myocardial morphogenesis.** (A) Model depicting the zebrafish embryonic wild-type heart during stages of cardiac cone formation and leftward jogging. At 15 hpf, bilateral populations of cardiomyocyte progenitor cells start converging towards the embryonic midline (black arrows). After 20 hpf, endocardial and myocardial progenitor cells initiate unilateral migrations towards the left, which causes a tilting of the heart cone and an elongation of the heart tube. (B-E) Maximum projections of confocal *z*-sections of 48 hpf zebrafish hearts. Unlike the looped wild-type heart (B), the *wnt9^DKO^* heart is collapsed and the atrium (highlighted by Myh6 staining) surrounds the ventricle (C). The ventricle fails to fuse anteriorly (white arrowhead; *n*=10/10 embryos analyzed). *wnt9b^sa20083^* homozygous; *wnt9a^sd49^* heterozygous mutants are phenotypically similar to *wnt9^DKO^* mutants (D; *n*=24/24 embryos analyzed), whereas *wnt9a^sd49^* homozygous;*wnt9b^sa20083^* heterozygous mutants have a wild-type phenotype (E; *n*=23/23 embryos analyzed). (F-I) Maximum projections of confocal *z*-scan sections with ventral views of the zebrafish heart field. At 20 hpf, wild-type cardiomyocyte progenitor cells have reached the midline and generated the heart cone (F). In *wnt9a*/*b* double morphants, anterior cardiomyocyte progenitor cells have not reached the embryonic midline and failed to fuse into the heart cone (G; *n*=10/10 embryos analyzed). At 24 hpf, cardiomyocyte progenitor cells have migrated towards the left side and generated an elongated heart tube (H). In *wnt9a*/*b* double morphants, cardiomyocyte progenitor cells have failed to fuse anteriorly and remain at the embryonic midline (I; *n*=10/10 morphants analyzed). The embryonic midline is marked with anti-ZO-1 staining. (J-M) Maximum projections of confocal *z*-scan sections with ventral views of endocardial progenitor cells. At 20 hpf, wild-type (J) and *wnt9a/b* double morphant (K) endocardial progenitor cells have reached the embryonic midline (*n*=6/6 morphants analyzed). At 24 hpf, endocardial progenitor cells have migrated towards the left side of the embryo and contribute to the elongating heart tube (L). In *wnt9a*/*b* double morphants, endocardial progenitor cells initiate a leftward movement (white arrowhead) but fail to complete leftward jogging (M; *n*=7/7 morphants analyzed). The embryonic midline is marked with anti-ZO-1 staining. (N-Q) Whole-mount *in situ* hybridization of *vmhc* cardiac expression during the stages of cardiac cone formation and leftward jogging. At 20 hpf, *vmhc* is expressed within the cardiac cone (N). In *wnt9^DKO^* mutants, expression of *vmhc* reveals the failure of the cardiac cone to close anteriorly (O; *n*=7/7 embryos analyzed). At 24 hpf, *vmhc* marks the wild-type heart tube during cardiac leftward jogging (P), whereas in *wnt9^DKO^* mutants, the heart remains at the embryonic midline (Q; *n*=7/7 embryos analyzed). White dashed lines indicate the embryonic midline. (R) Model depicting the zebrafish embryonic *wnt9^DKO^* heart during the stages of cardiac cone formation and leftward jogging. The bilateral population of cardiomyocyte progenitor cells fails to complete anterior migrations and instead forms a bilateral wing-like structure (black arrows), which fails to undergo leftward jogging. Scale bars: 30 µm.

During the formation of the nascent zebrafish heart tube, myocardial progenitor cells migrate from bilateral positions within the anterior lateral plate mesoderm towards the embryonic midline. This requires that these cells first migrate as coherent sheets of epithelia towards the embryonic midline. Then myocardial progenitor cells move in angular directions towards endocardial progenitor cells that have already arrived at the embryonic midline. Next, myocardial progenitor cells fuse at posterior positions, followed by a fusion at anterior positions. This generates a disc-shaped structure referred to as the ‘cardiac cone’ (Glickman Holtzman et al., 2007) (Fig. 1A). After remaining at the embryonic midline for several hours, cardiac progenitor cells undergo intricate morphological processes referred to as ‘cardiac tilting and jogging’. This transforms the flat mono-layered heart cone into the nascent heart tube, which extends towards the anterior and left (Bakkers, 2011; Rohr et al., 2008; Smith et al., 2008) (Fig. 1A; Movie 1). The angular movements of myocardial progenitor cells during heart cone formation are impaired in *npas4l* mutants that lack endocardial cells (Glickman Holtzman et al., 2007). This means that endocardial cells have some role in guiding their angular movements. Yet, it is still unclear which molecular mechanisms underlie this process and how the heart cone transforms into the nascent heart tube.

The growth of an embryo requires an intricate linkage between the positions of cells in tissues and their developmental activities. This is often regulated by morphogens, which are secreted proteins. Morphogens can move over significant distances within tissues to induce distinct responses among target cells in a concentration-dependent manner (Vincent and Briscoe, 2001). This occurs through an activation of signaling cascades and the activation or repression of transcription factors that determine the fate, morphology or behavior of cells. Morphogens can influence positional information and shape cells or tissues by regulating the contractile actomyosin system, adherens junctions or cell polarity (Gilmour et al., 2017). In many cases it is unclear how they shape tissues and organs, and whether cell fate decisions are involved.

Several secreted factors that act as morphogens in cardiac development have been identified, including the Wingless and Int-1 (Wnt) family of proteins (Akieda et al., 2019). The size of the nascent heart is sensitive to Wnt activity, as the overexpression of Wnt8 from a heat-shock transgene at pregastrula stages increases the size of the primary heart field in zebrafish (Ueno et al., 2007). Conversely, at later stages, Wnt8 inhibits the specification of cardiac progenitor cells. This gives Wnt8 biphasic roles, first as an inductive cue in early myocardial development before gastrulation and as a repressive signal afterwards. Overexpression studies during somitogenesis have established Wnt8 as being sufficient for promoting atrial cardiomyocyte differentiation while repressing ventricular cardiomyocyte proliferation (Dohn and Waxman, 2012; Ueno et al., 2007). Which endogenous Wnt ligands orchestrate morphogenetic events during early cardiac development has not been clarified. *In vitro*, the differentiation of cardiomyocytes from human pluripotent stem cells requires only a few signaling molecules, which include WNTs (Denning et al., 2016). This recapitulates the way cardiogenesis occurs during vertebrate development.

Downstream signaling initiated by Wnt ligands triggers the canonical β-catenin-dependent pathway or two non-canonical pathways called the planar cell polarity (PCP) or Ca^2+^-dependent pathways (Komiya and Habas, 2008). There are many known Wnt ligands, and these can activate exclusively only one of the pathways or trigger several (van Amerongen and Nusse, 2009), in a range of developmental contexts. Wnt11 activates PCP signaling during zebrafish cardiogenesis and a loss of either non-canonical pathways (PCP and calcium-dependent signaling) disrupts the migration of anterior endodermal progenitor cells towards the embryonic midline. Migrations of cardiac progenitor cells are also affected and result in cardia bifida, which is characterized by a bilateral localization of heart primordia (Matsui et al., 2005). PCP signaling positively regulates, via c-Jun N-terminal kinase (Jnk), the basic helix-loop-helix transcription factor Hand2, which plays an essential role in cardiac morphogenesis (Santos-Ledo et al., 2020). *wnt11* and *wnt5b* trigger PCP signaling during cardiac looping, which promotes the remodeling of the linear heart tube by polarizing actomyosin contractile networks (Merks et al., 2018).

Wnt9 proteins are ligands that have been implicated in both canonical and non-canonical signaling (Carroll et al., 2005; Goddard et al., 2017; Karner et al., 2009; Montcouquiol et al., 2006). During early stages of cardiac valvulogenesis in mice and zebrafish, *Wnt9b/wnt9b* expression is activated by the zinc-finger transcription factor Krüppel-like factor 2 (KLF2/Klf2), which has been induced by hemodynamic forces because of blood flow (Goddard et al., 2017). In mice, *Wnt9b* acts via the canonical pathway to block the proliferation of mesenchymal cells and mediate their condensation, which is required for the final steps of valve maturation (Goddard et al., 2017). A similar circuit is found in zebrafish, where blood flow also affects heart development via a Wnt9-dependent mechanism. Within the nascent zebrafish heart tube, the onset of blood flow induces the expression of *klf2a*/*b*, which in turn triggers the expression of two *wnt9a/b* paralogous genes within the endocardium. This Klf2-Wnt9 axis of mechanosensitive signaling is required for zebrafish cardiac valvulogenesis (Paolini et al., 2021).

Understanding the morphogenetic roles of specific Wnt signals during cardiac development has been difficult because multiple ligands and receptors are expressed at these stages (Ruiz-Villalba et al., 2016). In previous work, we demonstrated that one ligand, Wnt9a, plays a role in zebrafish cardiac valvulogenesis (Paolini et al., 2021). That study also uncovered potentially redundant and crucial roles of Wnt9a and its paralog, Wnt9b, during cardiac tube formation that were not further characterized.

Here, we demonstrate that Wnt9a/b play multiple roles during the assembly of the nascent heart cone and its transition towards the elongating heart tube. Our genetic studies show that the loss of both Wnt9 paralogs abolishes the completion of heart cone formation and leftward jogging of the heart tube. The simultaneous pharmacological inhibition of both canonical and non-canonical Wnt signaling pathways reproduces the *wnt9a/b* loss-of-function cardiac phenotype. This suggests that Wnt9a/b controls cardiac morphogenesis via divergent downstream signaling pathways. We also perform a broad misexpression experiment based on a heat-shock-inducible *wnt9b* transgene, which becomes expressed in all cell types. This reveals that Wnt9 is permissive but not instructive in guiding these early cardiac developmental processes. Finally, we provide evidence that Wnt9a/b prevent the premature differentiation of cardiac progenitor cells. These findings demonstrate that Wnt9 morphogens direct cardiogenesis through complex, interlinked signaling mechanisms.

## RESULTS

### Heart tube formation is severely impaired in *wnt9a*/*b* double mutants

To characterize the roles of Wnt9a and Wnt9b during cardiac tube formation, we generated *wnt9a^sd49^* (Grainger et al., 2016) and *wnt9b^sa20083^* (Sanger Institute Zebrafish Mutation Project Mutant Data Submission) double mutant embryos (hereafter referred to as *wnt9^DKO^* mutants) combined with a transgenic line harboring the myocardium-specific reporter Tg(*myl7:EGFP*)*^twu34^* (Huang et al., 2003). Strikingly, at 48 h postfertilization (hpf), *wnt9^DKO^* mutant hearts exhibited a collapsed form that was positioned at the embryonic midline. These hearts had apparently failed to undergo cardiac tilting and jogging, in contrast to wild-type hearts, and become arrested during early stages of heart morphogenesis (Fig. 1B,C; Movie 2). In all *wnt9^DKO^* mutant hearts, cardiac progenitor cells had failed to fuse in anterior positions (Fig. 1C, arrowhead; Movie 3) (*n*=10 mutants analyzed). This phenotype was similar to that seen in *npas4l* mutants, which lack the endocardium (Glickman Holtzman et al., 2007). The same phenotype occurred in *wnt9b^sa20083/sa20083^* homozygous; *wnt9a^sd49/+^* heterozygous mutants (Fig. 1D) (*n*=24/24 embryos analyzed). But *wnt9a^sd49/sd49^* homozygous; *wnt9b^sa20083/+^* heterozygous mutants had normally elongated hearts (Fig. 1E) (*n*=23/23 embryos analyzed). This genetic analysis showed that one wild-type allele of *wnt9b* is sufficient to rescue the cardiac phenotype, whereas only one wild-type allele of *wnt9a* does not. In contrast, none of the single mutants exhibited an early cardiac defect (Fig. S1A,B) (*n*=10/10 *wnt9a^sd49/sd49^* mutants analyzed; *n*=11/11 *wnt9b^sa20083/sa20083^* mutants analyzed).

To further verify these findings, we used an antisense oligonucleotide morpholino (MO) knockdown approach against Wnt9a/b. This produced a cardiac phenotype identical to that seen in *wnt9^DKO^* mutants at 48 hpf (Fig. S2). At 20 hpf, *wnt9a*/*b* double morphant heart cones failed to fuse anteriorly (*n*=15/15 embryos analyzed); consequently, they did not undergo cardiac jogging at 24 hpf (*n*=12/12 embryos analyzed). We applied counter-labeling with an antibody against the atrial cardiomyocyte-specific marker Myh6 (Yelon et al., 1999) at 48 hpf. This showed that in *wnt9a*/*b* double mutants, the collapsed myocardial ventricle was surrounded by atrial tissue in a cardiac cone-like arrangement (Movie 3). This demonstrated that the two *wnt9a*/*b* paralogous genes have essential and partially redundant roles during cardiac cone formation and leftward jogging and that Wnt9b plays a particularly important role in these processes.

Next, we characterized the morphologies of myocardial and endocardial progenitor cells at earlier stages (20-24 hpf). We used a MO knockdown against Wnt9a/b in different genetic backgrounds. First, we used the reporter transgene Tg(*nkx2.5:EGFP*)*^el83^*, which marks myocardial progenitor cells within the anterior lateral plate mesoderm and during their migration towards the embryonic midline (Witzel et al., 2012), which made it possible to visualize these cells at such an early stage. Another experiment involved the endothelium/endocardium-specific reporter transgene Tg(*etv2:GFP*)*^ci1^* (Proulx et al., 2010). We also performed whole-mount *in situ* hybridizations of wild-type and *wnt9^DKO^* mutants at 20, 24 and 36 hpf with probes against the chamber-specific genes *ventricular myosin heavy chain* (*vmhc*; also known as *myh7*) and *atrial myosin heavy chain* (*amhc*; *myh6*) (Yelon et al., 1999) (Fig. S3). At 20 hpf, wild-type myocardial and endocardial progenitors had fused into a heart cone at the embryonic midline (Fig. 1F,J,N; Fig. S3A,C). At this stage, both *wnt9a*/*b* double morphants (Fig. 1G; Fig. S2B,D) and *wnt9^DKO^* mutants exhibited myocardial progenitor cells arranged in two bilateral wings, marked by either EGFP or *vmhc* expression, that had not fused anteriorly (Fig. 1O) (*n*=32/32 morphants analyzed; *n*=7/7 mutants analyzed). This did not affect *etv2*:EGFP-positive endocardial progenitor cells, which originate in positions of the anterior lateral plate mesoderm and move towards the embryonic midline before the arrival of myocardial progenitor cells (Bussmann et al., 2007). These had already reached the embryonic midline in *wnt9a*/*b* double morphants, as seen in wild-type embryos (Fig. 1K; *n*=6/6 morphants analyzed).

By 24 hpf, wild-type myocardial and endocardial progenitors had formed an elongated heart tube that was jogging leftwards and towards the anterior (Fig. 1H,L,P; Fig. S3E,G). This starkly contrasted with the situation in *wnt9a*/*b* double morphants (Fig. 1I; Fig. S3F,H) and *wnt9^DKO^* mutants, where myocardial progenitor cells failed to complete the closure of the heart cone and remained at the embryonic midline (Fig. 1Q) (*n*=28/28 morphants analyzed; *n*=7/7 mutants analyzed). At this stage in *wnt9a*/*b* double morphants, *etv2:*EGFP-positive endocardial progenitor cells had initiated some leftward movements but failed to complete normal leftward jogging (Fig. 1M, white arrowhead; *n*=7/7 morphants analyzed). At 36 hpf, *vmhc-*expressing cells remained at the embryonic midline in these morphants, whereas in wild-type embryos the heart tube had extended towards the left (Fig. S3I,J). *amhc* had a similar expression: In *wnt9a*/*b* double morphants at 36 hpf, *amhc* expression had expanded symmetrically around the embryonic midline without the leftward- or anteriorly-directed displacement characteristic of wild-type embryos (Fig. S3K,L) (*n*=19/19 morphants analyzed).

*wnt9^DKO^* mutants exhibited impairments in heart tube formation and cardiac jogging, but the cardiac phenotypes differed strikingly from those observed in zebrafish mutants with defective left/right asymmetry signaling. In mutants lacking the nodal ligand Southpaw, the heart tube elongates along the embryonic midline in the anterior direction (Long et al., 2003). Next, we assessed whether left/right asymmetry signaling was affected in *wnt9^DKO^* mutants. We performed whole-mount *in situ* hybridizations at 20 hpf against the Nodal pathway gene *lefty1*, which is normally expressed at the embryonic midline and in a band to the left side of the heart field (Long et al., 2003). The expression of *lefty1* was similar in wild-type and *wnt9^DKO^* mutants (Fig. S4; *n*=20 embryos analyzed). This indicates that the paralogous genes *wnt9a* and *wnt9b* orchestrate early myocardial morphogenesis through effects on endocardial and cardiomyocyte progenitor cells that are independent of the signaling pathways that regulate left/right asymmetry (Fig. 1R).

### Wnt9b is permissive in controlling cardiomyocyte progenitor cell movements during heart cone formation

**Fig. 2.**
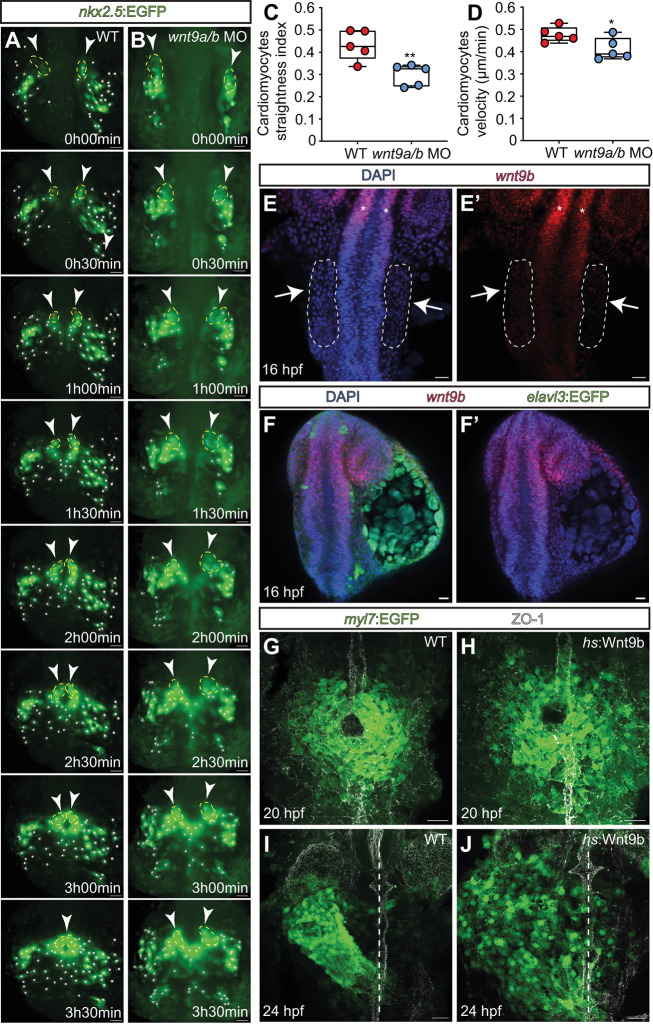
**Wnt9b has a permissive role during the anterior convergence movements and fusion of cardiomyocyte progenitor cells.** (A,B) Single images from light-sheet microscopy-derived time-lapse movies of cardiomyocyte progenitor cell migrations. Tracks of cardiac progenitor cell migrations during cardiac cone formation are highlighted. In the wildtype (A), cardiomyocyte progenitor cells migrate towards the embryonic midline and fuse first posteriorly and then anteriorly. In *wnt9a*/*b* double morphants (B), cardiomyocyte progenitor cells complete migrations and fuse posteriorly, but fail to converge anteriorly. Cardiomyocyte progenitor cells included in the tracking analysis (shown in C,D) have been marked. Dashed yellow lines and white arrowheads indicate the converging anterior part of the heart cone. All the other cell tracks are marked with white dots. (C,D) Tracking analyses of cardiomyocyte progenitor cells during cardiac cone formation. Quantification of the straightness index (C) and velocity (D) of wild-type versus *wnt9a*/*b* double morphant cardiomyocyte progenitor cells in anterior positions (wildtype, *n*=5 embryos; *wnt9a*/*b* double morphants, *n*=5 embryos). Box plots show mean values (middle bars) and first to third interquartile ranges (boxes). Lower and upper whiskers indicate minimum and maximum values, respectively (*P<0.05, **P<0.01; two-tailed, unpaired Student's *t*-test). (E-F′) Fluorescent whole-mount *in situ* hybridization of *wnt9b* expression at stage of cardiomyocyte progenitor cell convergence towards the embryonic midline. (E,E′) The lateral plate mesoderm (white arrows, dashed line) is devoid of *wnt9b* expression. Instead, *wnt9b* expression is in the neural tube (white asterisks) and neural retina (*n*=10/10 embryos analyzed). (F,F′) Neural progenitor cells expressing Tg*(elavl3:EGFP)^knu3^* do not co-express *wnt9b* (*n*=10/10 embryos analyzed). (G-J) Maximum projections of confocal *z*-scan sections with ventral views of cardiomyocyte progenitor cells. In wildtype, cardiomyocyte progenitor cells have fused into the heart cone (G). Upon heat shock-induced overexpression of *wnt9b*, the size of the heart cone increases (*n*=22/22 embryos analyzed) (H). At 24 hpf, wild-type cardiomyocyte progenitor cells undergo leftward cardiac jogging (I). Upon heat shock-induced overexpression of *wnt9b*, cardiomyocyte progenitor cells undergo leftward jogging but are more dispersed (J; *n*=13/13 embryos analyzed). The embryonic midline is marked by ZO-1 staining (G,H) and dashed lines (I,J). Scale bars: 40 µm (A,B); 30 µm (E-J).

Next, we characterized the morphogenetic dynamics of cardiac cone formation in *wnt9a*/*b* double morphants. Using the cardiac progenitor cell reporter transgene Tg*(nkx2.5:EGFP)^el83^*, we carried out longitudinal light-sheet microscopy observations *in vivo* at 17-22 hpf. In the wild-type, cardiomyocyte progenitors migrated from positions in the lateral plate mesoderm towards the embryonic midline where the cardiac cone fused. The cardiac cone first fused in posterior positions before fusing anteriorly (Fig. 2A; Movie 4). The cone also fused at the posterior in *wnt9a/b* double morphants but failed to complete anterior fusion (Fig. 2B; Movie 5). For a more detailed analysis of the migratory behavior of cardiomyocytes, we used the longitudinal movie files to reconstruct individual cell tracks (Fig. 2A,B; Fig. S5; Movies 4, 5). Here we assessed both cell velocities and the straightness index, which is used to measure the direction of migration paths (whereby 1 equals a straight line). Our analysis revealed that the loss of Wnt9a/b reduced the velocity of cells and disrupted the straightness of their paths (Fig. 2C,D; Fig. S5; Movies 4,5; *n*=5 each of *wnt9a*/*b* double morphant and wild-type hearts analyzed). This demonstrates that Wnt9a/b play an important role in the migrations of cardiac progenitor cells during cardiac cone formation and jogging.

These results prompted us to check the expression pattern of *wnt9b* in the wild-type*.* We did so using whole-mount *in situ* hybridizations at 16 hpf. At this stage, cardiac progenitor cells in bilateral positions of the anterior lateral plate mesoderm initiate migrations towards the embryonic midline (Bakkers, 2011; Glickman Holtzman et al., 2007). We found that the anterior lateral plate mesoderm was devoid of *wnt9b* expression at this stage (Fig. 2E,E′, see arrows; Movie 6; *n*=10 embryos analyzed). It was expressed, however*,* in the anterior and ventral sides of the neural tube (Fig. 2E,E′; Movie 6). At this stage, the anterior neural tube is made up mostly of neural progenitors, as indicated by a weak expression of the post-mitotic neuronal marker transgene Tg*(elavl3:EGFP)^knu3^* (Park et al., 2000). *wnt9b* was never expressed in cells that have developed to the point that they express this reporter transgene (Fig. 2F,F′; Movies 7,8; *n*=10 embryos analyzed). It is not produced in the population of postmitotic neurons located at the anterior/ventral side of the neural tube. Instead, *wnt9b* is expressed by neural progenitors at the ventral midline of the neural tube at the time of cardiac cone formation.

The finding that *wnt9b* is not expressed within cardiac progenitor cells, but rather in neighboring cells at the ventral midline of the neural tube, suggested that the signal plays an instructive role during cardiac cone formation. To test this possibility, we generated the transgenic line Tg*(hsp70l:wnt9b_IRES_EGFP)^pbb48^*, which causes an embryo-wide overexpression of *wnt9b* (Fig. S6A) and causes an activation of the canonical Wnt signaling reporter Tg*(7xTCF-Xla.Siam:nlsmCherry)^ia5^* throughout the heart (Fig. S6B,C). When heat-shocked at 3.5 hpf, Tg*(hsp70l:wnt9b_IRES_EGFP)^pbb48^* embryos developed with a dorsalized phenotype (Fig. S6D,E). If Wnt9b played an instructive role, misexpression ought to disrupt the unidirectional migration patterns of myocardial progenitor cells. The application of a heat shock at 14 hpf (30 mins at 37°C) led to an increase in the size of the cardiac cone in *wnt9b*-overexpressing embryos at 20 hpf compared with the wild-type (Fig. 2G,H; *n*=22 embryos analyzed). The increase did not, however, impair either heart cone formation or the leftward-directed jogging of the nascent heart tube at 24 hpf. Myocardial progenitor cells had moved to the left side from the midline, but they were more widely dispersed than in the wild-type (Fig. 2I,J; *n*=13 Wnt9b-overexpressing embryos analyzed). As a result, Wnt9b is not instructive but is rather permissive during heart cone formation and cardiac jogging.

### The paralogous *wnt9a*/*b* genes have canonical and non-canonical roles during cardiac cone formation

**Fig. 3.**
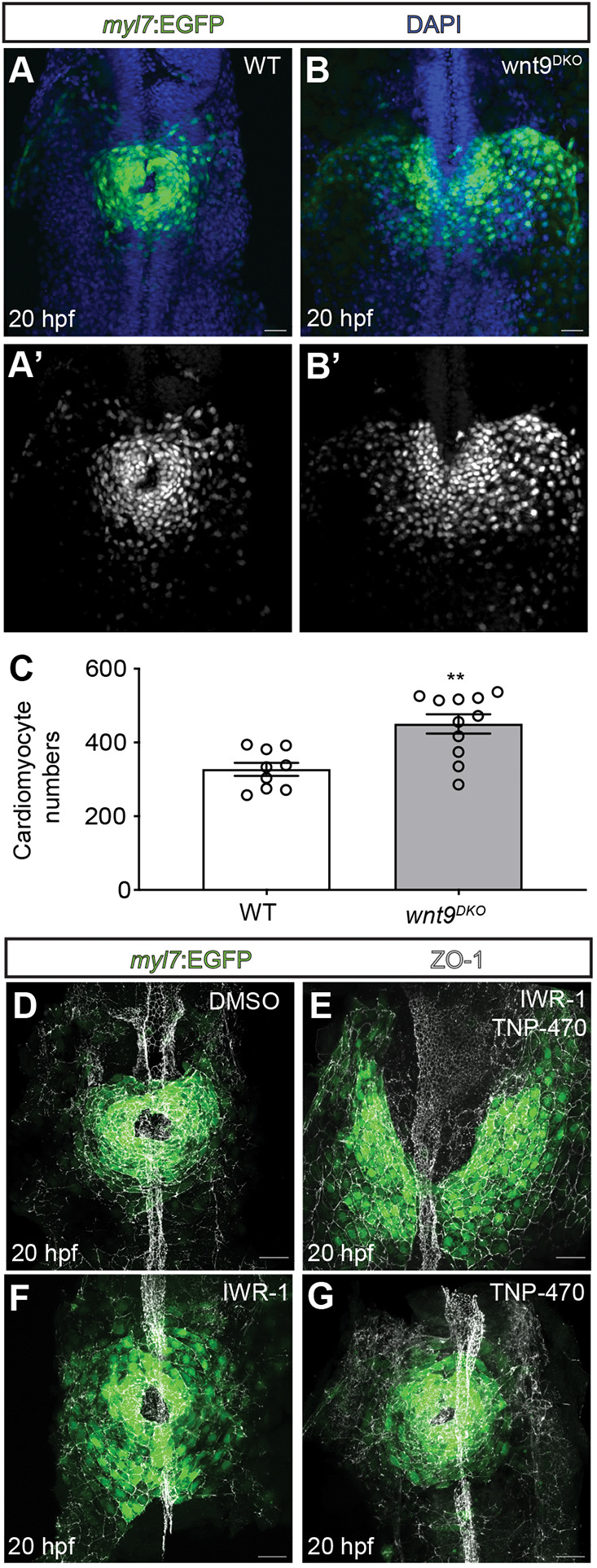
**Wnt9a/b signal via canonical and non-canonical pathways during cardiac cone formation.** (A-C) Quantification of cardiomyocyte numbers in wildtype versus *wnt9^DKO^* mutants during cardiac cone formation. Cardiomyocyte numbers increase in *wnt9^DKO^* mutants (wildtype, *n*=9 embryos; *wnt9^DKO^*, *n*=11 embryos). Data are mean±s.e.m. Dots represent single values (**P<0.01; two-tailed, unpaired Student's *t*-test). (D-G) Maximum projections of confocal *z*-scans with ventral views of cardiomyocyte progenitor cells during cardiac cone formation. In DMSO-treated control embryos, the heart cone has formed by 20 hpf (D) whereas in IWR-1- and TNP-470-treated embryos, cardiomyocyte progenitor cells have not reached the embryonic midline and failed to fuse into a heart cone (E; *n*=21/21 embryos analyzed). Heart cones of embryos treated only with IWR-1 (F; *n*=6/6 embryos analyzed) or TNP-470 (G; *n*=11/11 embryos analyzed) have no anterior fusion defects. The treatment with IWR-1, TNP-470, their combination or DMSO (control) was carried out from 14 to 20 hpf. The embryonic midline is labeled for ZO-1. Scale bars: 30 µm.

Canonical Wnt signaling in the pre-cardiac mesoderm is necessary and sufficient to inhibit cardiac progenitor cell differentiation (Dohn and Waxman, 2012; Ueno et al., 2007). We confirmed this when observing an increase in cardiomyocyte numbers in *wnt9^DKO^* mutants at 20 hpf compared with the wild-type (Fig. 3A-C; *n*=9 wild-type and *n*=11 *wnt9^DKO^* mutant embryos analyzed). However, the severe morphogenetic changes observed in *wnt9^DKO^* mutants could not be explained only by a lack of canonical Wnt activity. This can be seen from embryos that overexpress the Wnt antagonist Dkk1; upon a loss of canonical Wnt signaling after gastrulation, zebrafish still undergo heart cone formation and leftward cardiac jogging (Dohn and Waxman, 2012; Ueno et al., 2007). In *wnt11* (Matsui et al., 2005) or *wnt11r* (Choudhry and Trede, 2013) mutants, which inhibit non-canonical Wnt signaling, zebrafish still form a leftward extending heart tube. This suggested that Wnt9a/b signaling triggers the activation of a combination of canonical and non-canonical Wnt pathways during zebrafish cardiac morphogenesis.

To test this hypothesis, we treated zebrafish embryos with IWR-1, an inhibitor of the canonical Wnt pathway (Lu et al., 2009) in combination with TNP-470, which inhibits the non-canonical Wnt pathway (Zhang et al., 2006). IWR-1 abolishes the turnover of the Axin protein; this strongly decreases levels of β-catenin, a central component of canonical Wnt signaling (Chen et al., 2009). TNP-470 targets Methionine aminopeptidase 2, which acts downstream of Frizzled receptors and upstream of the non-canonical Wnt pathway components Dishevelled, JNK and CaMKII (Zhang et al., 2006). Treatment with both small compound inhibitors from 14 to 20 hpf prevented the anterior fusion of the cardiac cone (Fig. 3D,E; *n*=21 embryos analyzed). This resembled the phenotype of *wnt9^DKO^* mutants (Fig. 1O). In comparison, separate treatments with single inhibitors did not produce an early cardiac cone phenotype (Fig. 3F,G; *n*=11 embryos analyzed). These findings suggest that signaling via Wnt9a/b leads to the activation of both canonical and non-canonical Wnt signaling pathways during cardiac cone formation and jogging, through a complex regulation of several downstream Wnt components.

### The control of Wnt9a/b cardiac cone formation and jogging is independent of apico-basal cell polarity

**Fig. 4.**
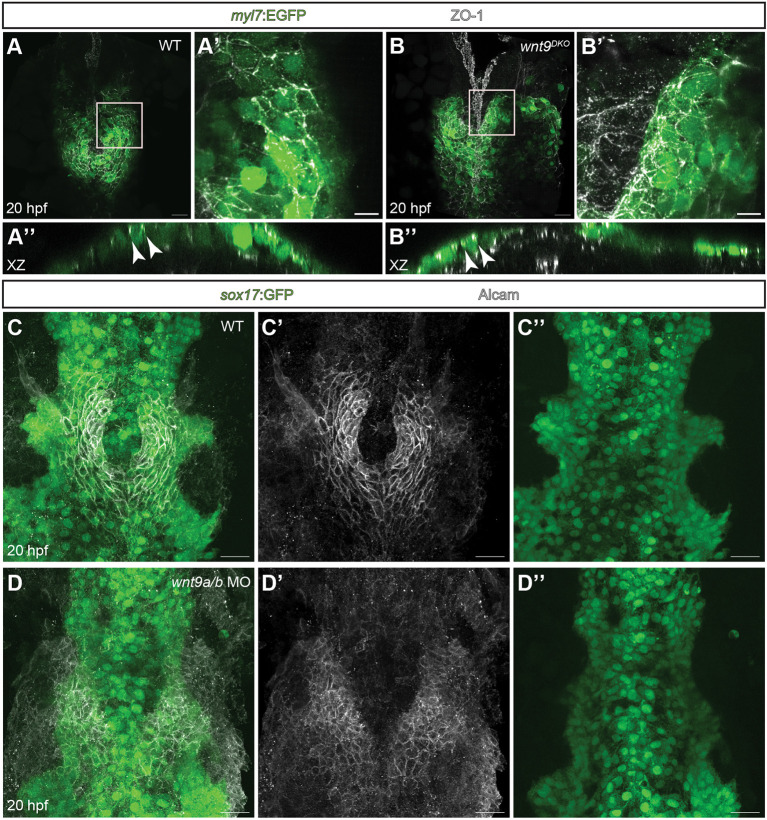
**Wnt9a/b control cardiac cone formation and leftward jogging independently of epithelial cell polarity or endoderm.** (A-B″) Maximum projections of confocal *z*-scans with ventral views (A,A′ and B,B′) or *x*-*z* section views (A″,B″) of the zebrafish heart field. Maximum projection of a 7 µm deep *z*-stack showing subapical tight junctional ZO-1 staining within the myocardium at 20 hpf (A′). *x*-*z* views (A″) show the correct subapical localization of tight junctions between cardiomyocytes (white arrowheads; *n*=9/9 embryos analyzed). In *wnt9^DKO^* mutants, apico-basal cell polarity is intact, as indicated by a maximum projection of confocal *z*-scans (B) or of a 7 µm deep *z*-stack (B′), which reveals intact tight junctions. *x*-*z* view (B″) of the *wnt9^DKO^* heart field which shows intact tight junctions within the myocardium (white arrowheads; *n*=12/12 embryos analyzed). (C-D″) Maximum projections of confocal *z*-scans with ventral views of cardiomyocyte progenitor cells during cardiac cone formation and of the underlying endoderm. In the wildtype (C-C″), the endoderm is present at the midline while the cardiac cone is completing its fusion. In *wnt9a*/*b* double morphants (D-D″), the endoderm is present at the embryonic midline, whereas the anterior portion of the cardiac cone does not migrate towards the embryonic midline and fails to fuse into the heart cone (*n*=15/15 embryos analyzed). Scale bars: 30 µm (A,B,C-D″); 10 µm (A′,B′).

Apicobasal polarity is crucial to heart morphogenesis. By the 15-somite stage, cardiomyocyte progenitor cells have formed polarized epithelial sheets that migrate towards the embryonic midline (Rohr et al., 2006). Loss of either Protein kinase C iota (Prkci) or MAGUK p55 subfamily member 5 (Mpp5; Pals1a), proteins which establish apicobasal polarity, disrupts epithelial organization, and cardiac morphogenesis stalls at the heart cone stage (Horne-Badovinac et al., 2001; Rohr et al., 2006). This produces defects in cardiac cone formation and leftward jogging that strongly resemble *wnt9^DKO^* mutant cardiac phenotypes (Fig. 1O) (Horne-Badovinac et al., 2001; Rohr et al., 2006). Conceivably this situation in *wnt9^DKO^* mutants might be due to a disruption of the apico-basal polarity of cardiomyocyte progenitors. To determine whether this was the case, we analyzed the expression of the protein Zonula occludens-1 (ZO-1; Tjp1a), which is a marker of apical tight junctions (Fig. 4A-B′; *n*=9 wild-type and *n*=12 mutant embryos analyzed). The subcellular distribution of ZO-1 was similar in wild-type and *wnt9^DKO^* mutants (Fig. 4A″,B″, see arrowheads). This suggested that the loss of the two *wnt9a*/*b* paralogous genes did not affect the apico-basal polarity of cardiomyocytes.

Genetic studies have shown that the migration of myocardial progenitor cells toward the midline and the formation of the cardiac cone depends on the presence of endoderm. In zebrafish mutants lacking endoderm, cardiac progenitor cells fail to migrate toward the embryonic midline, which results in cardia bifida (Dickmeis et al., 2001; Fukui et al., 2014). This prompted us to assess the organization of endodermal progenitor cells in *wnt9a*/*b* double morphants. We did this using the transgenic endodermal reporter line Tg(*sox17:GFP*)*^s870^* (Field et al., 2003), and counter-labeled myocardial tissue with an antibody against Activated leukocyte cell adhesion molecule (Alcam) (Beis et al., 2005) (Fig. 4C-D″). This revealed that at 20 hpf, endodermal tissue at the embryonic midline was similar in the double morphants and wild-type embryos (Fig. 4C-D″; *n*=15/15 morphants analyzed).

The extracellular matrix glycoprotein Fibronectin (Fn) is deposited between the endoderm and cardiac precursors at the embryonic midline and it is essential for the timely migration of cardiomyocyte progenitors (Trinh and Stainier, 2004). To assess whether the absence of the Wnt9 paralogs alters Fn deposition, we assessed Fn expression at heart cone stages in *wnt9^DKO^* in comparison with wild-type embryos. High-resolution *x*-*z* scan projections through different section planes of the heart cone revealed no qualitative differences in Fn deposition between these conditions (Fig. S7; *n*=7/7 mutants analyzed). Therefore, Fn deposition is not directly affected or causative to the *wnt9^DKO^* cardiac phenotype. These findings suggested that Wnt9a/b control myocardial progenitor cell migrations and heart cone assembly while not disrupting endodermal midline migration and fusion. Furthermore, the loss of these proteins affected myocardial progenitor cell migrations without causing any obvious defects in epithelial apico-basal polarity or Fn deposition.

### Wnt9a/b activity in the heart cone does not depend on Klf2 or endocardium

**Fig. 5.**
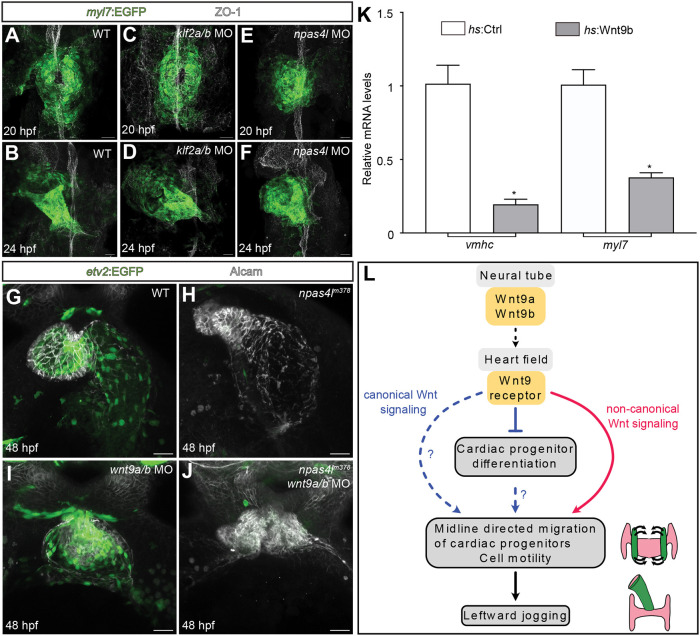
**The activity of Wnt9a/b does not depend on the endocardium and prevents premature cardiac differentiation in the heart cone.** (A-F) Maximum projections of confocal *z*-scans with ventral views of cardiomyocyte progenitor cells. In the wildtype (A), cardiomyocyte progenitor cells complete fusion into the heart cone by 20 hpf and undergo leftward jogging by 24 hpf (B). In *klf2a/b* double morphants (C), cardiomyocyte progenitor cells are not defective during the fusion of the cardiac cone (*n*=10/10 morphants analyzed) or during leftward jogging (D; *n*=9/9 morphants analyzed). In *npas4l* morphants (E), which lack all endocardial cells, cardiomyocyte progenitor cells do not fuse correctly at the embryonic midline (*n*=8/8 embryos analyzed). Yet, the heart cone undergoes leftward jogging (F; *n*=10/10 embryos analyzed). (G-J) Maximum projections of confocal *z*-scans of 48 hpf zebrafish hearts. Different to the looped wild-type heart (G), the *npas4l* morphant heart (H) is strongly ballooned. In comparison, the *wnt9a*/*b* double morphant mutant heart (I) is collapsed. The knockdown of *wnt9a*/*b* in *npas4l^m378^* mutants produces the *wnt9a*/*b* double morphant cardiac phenotype with a collapsed heart (J; *n*=30/30 embryos analyzed). This suggests that the *wnt9a*/*b* morphant cardiac phenotype is not due to endocardial defects. (K) Quantifications of changes of mRNA expression levels of myocardial differentiation markers by quantitative real-time PCR. Shown is the comparison in Tg*(hsp70l:wnt9b_IRES_EGFP)^pbb48^* transgenic embryos with their wild-type heat-shocked siblings (*n*=4 experiments; *P<0.05; two-tailed, paired Student's *t*-test). (L) Model depicting how canonical and non-canonical signaling by Wnt9a/b may affect the midline-directed migration of cardiomyocyte progenitor cells, formation of the cardiac cone and leftward jogging. Scale bars: 30 µm.

In endocardial cells, *wnt9a*/*b* are induced by blood flow and Klf2, and their expression is required for cardiac valvulogenesis (Goddard et al., 2017; Paolini et al., 2021). This raised the question of whether the activity of Wnt9a/b during heart cone formation requires Klf2. To determine this, we examined whether *klf2a*/*b* double morphants affected the development of the heart cone or cardiac jogging. At 20 hpf, the heart cone of *klf2a*/*b* double morphants was fused at the embryonic midline (Fig. 5C; *n*=10/10 embryos analyzed) as in the wild-type (Fig. 5A). By 24 hpf, the heart tube was elongating towards the left (Fig. 5D; *n*=9/9 embryos analyzed), again resembling the wild-type (Fig. 5B). This confirms the finding that *klf2a*/*b* double mutants develop an elongated heart tube (Fontana et al., 2020) and distinguishes them from *wnt9^DKO^* mutants at the heart cone and jogging stages (Fig. 1G,I). At this point, the production of Wnt9a/b does not appear to be contingent upon the activity of Klf2a/b.

Conceivably, Wnt9a/b activity might depend on the endocardium itself, which fails to form in early zebrafish embryos lacking Npas4l (Reischauer et al., 2016) However, the phenotype of *npas4l* zebrafish is quite different from those lacking Wnt9a/b (Fig. 1G,I) (Reischauer et al., 2016; Glickman Holtzman et al., 2007) and heart cone formation proceeds normally at 20 hpf (Fig. 5E; *n*=8/8 embryos analyzed), as does cardiac jogging towards the left at 24 hpf (Fig. 5F; *n*=10/10 embryos analyzed). To further establish whether Wnt9a/b signaling for cardiac progenitor cell migration involved the endocardium, we performed a genetic epistasis experiment by knocking down Wnt9a/b in *npas4l^m378^* mutants. Although *npas4l^m378^* mutants had an enlarged myocardial heart tube at 48 hpf (Fig. 5H) compared with the wild-type heart (Fig. 5G), the myocardium of *npas4l^m378^* mutant; *wnt9a*/*b* double morphants was collapsed (Fig. 5J; *n*=30/30 embryos analyzed). This resembled the phenotype of *wnt9a*/*b* double mutants (Fig. 1C) and morphants (Fig. 5I). This shows that the loss of Wnt9a/b is epistatic over the *npas4l* mutant phenotype, and thus rules out an involvement of the endocardium in *wnt9a*/*b*-mediated signaling during cardiac cone formation.

### Wnt9 signaling prevents the premature differentiation of myocardial progenitor cells

Another way Wnt9 might affect cardiac morphogenesis is by forcing the premature differentiation of myocardial progenitor cells. Canonical Wnt signaling has a role in inhibiting cardiac progenitor differentiation (Dohn and Waxman, 2012; Ueno et al., 2007). This is in line with our observation that *wnt9^DKO^* mutants had more cardiomyocyte progenitor cells (Fig. 3A-C). The differentiation state of these cells could affect their motility and alter the development of the tissue. Next, we determined whether Wnt9b affected the differentiation states of myocardial progenitors. To do this, we examined levels of the expression of developmental marker genes in myocardial cells, comparing Tg*(hsp70l:wnt9b_IRES_EGFP)^pbb48^* transgenic embryos, which overexpressed Wnt9b, with their wild-type heat-shocked siblings. Upon heat-shock at 14 hpf, the differentiation of myocardial progenitor cells at 20 hpf was impaired (Fig. 5K). This is in line with an established role of canonical Wnt signaling in preventing excessive cardiac differentiation during stages of heart cone formation.

## DISCUSSION

How morphogen signaling controls tissue and organ shape is a major problem of developmental biology. Secreted Wnt family molecules act as morphogens in many developmental and pathological processes. Wnt9a/b contribute to cardiac development when they are induced by blood flow and have essential roles during valvulogenesis (Goddard et al., 2017; Paolini et al., 2021). Loss of *wnt9a* results in a complete lack of valve leaflets in zebrafish (Paolini et al., 2021) and loss of *Wnt9b* causes an enlarged and malfunctioning atrioventricular valve in mice (Goddard et al., 2017). Here, we demonstrate that the *wnt9a/b* paralogous genes play an equally crucial role earlier, during the first stages of heart development in zebrafish.

Our findings demonstrate that Wnt9-mediated signaling is necessary for the formation of the cardiac cone of the nascent zebrafish heart and requires signaling via both canonical and non-canonical pathways (Fig. 5L). We made a closer investigation to determine the extent to which the effects of the two ligands and the pathways can be distinguished. We find that these two branches of Wnt signaling have partially redundant effects on myocardial progenitor cells that were uncovered only through an examination of *wnt9^DKO^* mutants or by pharmacologically inhibiting both pathways. We show that a loss of Wnt9a/b causes the premature differentiation of myocardial progenitor cells and that their migration toward the embryonic midline is disrupted. These two phenotypes might be connected as differentiated cells tend to lose their motile capacity. This is the case during zebrafish cardiac regeneration, when differentiated cardiomyocytes first undergo dedifferentiation, then become motile and migrate to injured regions of the heart (Itou et al., 2012; Kikuchi et al., 2010).

Inhibiting only canonical Wnt pathway signaling is not sufficient to explain the defective cardiac progenitor cell migration in *wnt9^DKO^* mutants. This suggests that non-canonical Wnt signaling must also be involved in orchestrating convergence movements of anterior myocardial progenitor cells towards the embryonic midline. This could be because non-canonical signaling affects the cytoskeletal organization of anterior myocardial progenitor cells during their migration towards the midline, but this will require further investigation. Another possibility is that the defects observed in *wnt9^DKO^* mutants arise because Wnt9 affects the apico-basal polarity of cardiomyocyte progenitor cells in some way that has escaped detection so far.

These observations suggest that canonical and non-canonical Wnt pathways cooperate to direct the convergence of myocardial progenitor cells towards the embryonic midline. In contrast, the migration of endocardial progenitor cells is not affected in *wnt9^DKO^* mutants. This suggests that Wnt9a/b signaling exerts a specific control on myocardial progenitor cell migrations at a crucial stage in heart development.

The defects in migration that we observe in *wnt9^DKO^* mutants may be due to defects in the organization of the actin cytoskeleton. The effectors of this system are controlled by Frizzled/PCP signaling, activated by non-canonical Wnt ligands (Gray et al., 2011). During sprouting angiogenesis, low levels of non-canonical Wnt signaling disrupt collective migrations of endothelial cells. This is caused by a weakening of adherens junctions, which couples the force transmission from neighboring cells or the extracellular matrix towards the actin cytoskeleton (Carvalho et al., 2019). Hence, a disruption of cytoskeletal organization could slow down myocardial progenitor cells during their migration towards the midline. It remains unclear why the lack of the Wnt9 paralogs specifically affects the migration of myocardial progenitors at the anterior of the heart cone. Future work will determine whether cells at the anterior express distinct Wnt9 receptors.

Our genetic studies demonstrate that *wnt9b* plays a particularly important role during early cardiac morphogenesis. At heart cone stages, its expression is absent from the lateral plate mesoderm, but present within the ventral neural tube. This suggests that during heart cone assembly, *wnt9b* is produced in the ventral part of the neural tube and spreads towards the heart field, affecting the dynamics of myocardial progenitors in a paracrine manner. In other contexts, Wnt ligands play a role in coordinating the development of neural and vascular tissues. For example, in the developing nervous system, Wnt7a/b coordinate neural tube and retina vascularization (Paredes et al., 2018; Peguera et al., 2021). Neural tissues also secrete Wnt7a/b to activate Wnt signaling in endothelial cells during the vascularization of the spinal cord (Daneman et al., 2009). *In vitro* studies have shown that the development of cardiomyocytes is influenced by neural tissue, as cardiomyocytes and neurons develop concurrently from embryonic stem cells, suggesting bidirectional communication between the two cell types (Murashov et al., 2005).

Single genes frequently play roles in the development of several traits and contribute to diverse pathologies. Here, we show how *wnt9a* and, in particular, *wnt9b* contribute to heart morphogenesis before the onset of blood flow. This complements earlier studies, which demonstrated that Wnt9a and Wnt9b act later, in a flow-dependent manner, to sculpt heart valves (Goddard et al., 2017; Paolini et al., 2021). It is known that pleiotropic congenital heart defects (CHD) may arise from the same gene defect (Paaby and Rockman, 2013). These studies hint that Wnt9a/b and blood flow may contribute to CHDs. Further studies are needed to determine whether mutations in human *WNT9A*/*B* paralogous genes are associated with CHDs.

## MATERIALS AND METHODS

### Zebrafish

Handling of zebrafish was carried out according to FELASA guidelines and in compliance with German and Brandenburg state law, carefully monitored by the local authority for animal protection (LAGV, Brandenburg, Germany; Animal protocol #2347-18-2015). The following strains of zebrafish were maintained under standard conditions as previously described (Westerfield et al., 1997): *wnt9a^sd49^* (Grainger et al., 2016), *wnt9b^sa20083^* (Sanger Institute Zebrafish Mutation Project Mutant Data Submission), *npas4l^m378^* (Reischauer et al., 2016), Tg*(myl7:EGFP)^twu34^* (Huang et al., 2003), Tg*(nkx2.5:EGFP)^el83^* (Witzel et al., 2012), Tg*(etv2:GFP)^ci1^* (Proulx et al., 2010), Tg*(elavl3:EGFP)^knu3^* (Park et al., 2000), Tg*(hsp70l:wnt9b_IRES_EGFP)^pbb48^* (this study), Tg*(sox17:GFP)^s870^* (Field et al., 2003), Tg*(7xTCF-Xla.Siam:nlsmCherry)^ia5^* (Moro et al., 2012). The developmental stage of the embryos used is indicated for each experiment in the results and figure legends.

### Antisense MO injections

The following MOs were injected into the yolk at the one-cell stage in 1 nl total volume: *wnt9a* (5′-AAGAATTGTCCTGCCTACCCGAAGT-3′) (1 ng/embryo) (Paolini et al., 2021), *wnt9b* (5′-ACCTGTAAGCCTAACGAAAACACAA-3′) (1 ng/embryo) (Jackson et al., 2015), *klf2a* (5′-CTCGCCTATGAAAGAAGAGAGGATT-3′) (1 ng/embryo) (Nicoli et al., 2010), *klf2b* (5′-AAAGGCAAGGTAAAGCCATGTCCAC-3′) (5 ng/embryo) (Renz et al., 2015), *cloche/npas4l* (5′-GAGTCTCCGCAGCTCATCTCACA-3′) (1 ng/embryo) (Reischauer et al., 2016). Control embryos (WT) were always injected with 1 nl of water.

### Generation of Tg*(hsp70l:wnt9b_IRES_EGFP)^pbb48^*

The open reading frame of zebrafish *wnt9b* (ENSDARG00000037889) was amplified by PCR with AK3 and AK4 primers (Table 1) and cloned into the Gateway *pDONR221* vector via Gateway BP cloning (Thermo Fisher Scientific) to generate *pME-Wnt9b*. The pDest_Tol2pA_*hsp70l:wnt9b_IRES_EGFP* vector was created by Gateway LR cloning (Thermo Fisher Scientific) from *pDest_Tol2pA*, *p5E-hsp70l*, *pME-Wnt9b* and *p3E-IRES-eGFPpA*. We injected 15 pg of plasmid into one-cell-stage embryos with 15 pg of *Tol2* transposase mRNA.

**Table 1.**
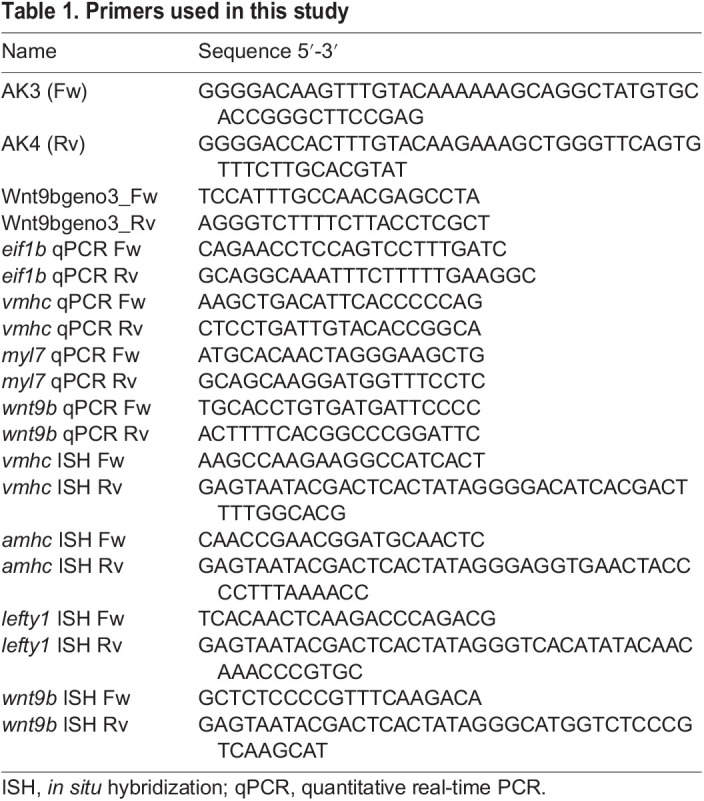
Primers used in this study

### Genotyping of *wnt9b^sa20083^* mutants

*wnt9b^sa20083^* mutants were genotyped through PCR amplification of the coding region containing the point mutation (with Wnt9bgeno3_FW and Wnt9bgeno3_RV primers, see Table 1) and enzymatic digestion of the amplified fragment (234 bp) was carried out using *NaeI* (New England Biolabs). The mutated fragment does not contain the restriction site and was not cut.

### Heat shock and chemical treatments

To overexpress *wnt9b* during the stages of heart cone fusion and leftward jogging, transgenic fish carrying Tg*(hsp70l:wnt9b_IRES_EGFP)^pbb48^* were crossed to Tg*(myl7:EGFP)^twu34^* transgenic fish and the resulting embryos were heat-shocked at 3.5 or 14 hpf (30 min at 37°C) and screened for EGFP expression. To inhibit canonical Wnt signaling, embryos were treated with 20 µM IWR-1 (Selleckchem) between 15 and 20 hpf. To inhibit non-canonical Wnt signaling, embryos were treated with 50 µM TNP-470 (Sigma-Aldrich) between 15 and 20 hpf. Control embryos were always treated with an equivalent amount of DMSO. To inhibit pigmentation, embryos were treated with 1-phenyl-2-thiourea (PTU) (Sigma-Aldrich) after 24 hpf.

### Quantification of mRNA expression by RT-qPCR

For RT-qPCR experiments, 25 heat-shocked EGFP-positive Tg*(hsp70l:wnt9b_IRES_EGFP)^pbb48^* embryos were pooled. Similarly, 25 heat-shocked EGFP-negative siblings (three biological replicates) were pooled. Total RNA was extracted using Trizol (Thermo Fisher Scientific) and Phase Lock Gel Heavy tubes (1.5 ml, 5 Prime) and the corresponding cDNA was synthesized from total RNA with the RevertAid H Minus First Strand cDNA Synthesis kit (Thermo Fisher Scientific). RT-qPCR experiments were performed as previously described (Renz et al., 2015) using 9 ng cDNA per technical replicate and the KAPA Sybr Fast qPCR kit (Roche) on an Analytic-Jena qTOWER 3 (Analytic Jena, 844-00553-2). Cycle threshold (Ct) values were determined by Analytic Jena qPCRsoft (Analytic Jena, version 4.1.3.0). *eif1b* was used as a housekeeping gene for normalization. *wnt9a*/*b* morphant sample values were normalized to 1, using the comparative threshold cycle method (2-DDCT) (Livak and Schmittgen, 2001). As every single biological replicate represents an independent experiment from an independent clutch of embryos, two-tailed, paired *t*-tests were performed using GraphPad Prism (version 9). For the experiment in Fig. 5K, the mean values of the fold changes are indicated in Table 2. The mean values of fold changes for Fig. S6A are indicated in Table 5. All primers are listed in Table 1.

**Table 2.**
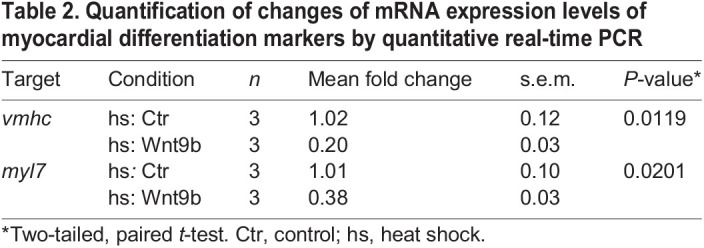
Quantification of changes of mRNA expression levels of myocardial differentiation markers by quantitative real-time PCR

### Whole-mount *in situ* hybridization, immunohistochemistry and image acquisition

Zebrafish embryos were collected at 20 hpf or 24 hpf and fixed with 4% paraformaldehyde overnight at 4°C. The *vmhc*, *amhc*, *lefty1* and *wnt9b* antisense *in situ* probes were generated by PCR amplification from 24 hpf WT cDNA, with the reverse primers containing a T7 promoter overhang sequence (primers listed in Table 1). Antisense RNA was synthesized using the DIG RNA Labeling kit (Roche) and T7 polymerase. Whole-mount *in situ* hybridization was performed as previously described (Jowett and Lettice, 1994). For fluorescence *in situ* hybridization experiments, embryos were treated with 2% H_2_O_2_ in PBS for 10 min to quench endogenous peroxidase. For signal detection, embryos were incubated in 1:300 cyanine 3 amplification reagent (PerkinElmer) diluted in 1× plus amplification diluent (PerkinElmer). Brightfield images were recorded with 20× objectives on an Axioskop (Zeiss) with an EOS 5 D Mark III (Canon) camera and processed using Adobe Camera Raw and Adobe Photoshop (Adobe Creative Cloud). Fluorescence images were recorded on an LSM 880 confocal microscope (Zeiss) and processed with Fiji (Schindelin et al., 2012) in order to minimize the background and highlight the signal.

Whole-mount immunohistochemistry was performed on 20, 24 and 48 hpf embryos. The following primary antibodies were used: mouse anti-Zn-8/Alcam (1:25, Developmental Studies Hybridoma Bank, zn-8, AB_531904), mouse anti-Myh6 (1:10, Developmental Studies Hybridoma Bank, AB_528376), mouse anti-ZO-1 (1:200, Thermo Fisher Scientific, 33-9100), rabbit anti-Fibronectin (1:100, Sigma-Aldrich, F3648). The secondary antibodies used were Alexa Fluor 633-conjugated goat anti-mouse (1:200, Thermo Fisher Scientific, A-21052), HRP-conjugated goat anti-rabbit (1:1, Thermo Fisher Scientific, from Tyramide SuperBoost kit, B40958). Embryos were fixed with 4% paraformaldehyde overnight. For Zn-8/Alcam staining, embryos were permeabilized with ice-cold acetone for 10-15 min at −20°C. After 1 h of incubation in blocking solution [PBST (PBS with 0.1% Tween20) with 1% DMSO and 5% normal goat serum (NGS)], embryos were incubated with primary antibody diluted in PBST with 1% DMSO and 1% NGS. For Myh6 staining, embryos were incubated for 2 h in blocking solution [PBST with 10% NGS, 2 mg/ml bovine serum albumin (BSA) and 0.2% saponin] and then with primary antibody diluted in PBST with 0.2% saponin. For ZO-1 staining, embryos were incubated for 2 h in blocking solution (PBST with 10% NGS, 2 mg/ml BSA and 0.8% Triton X-100) and then with the primary antibody in PBST and 0.8% Triton X-100. For Fn staining, embryos were incubated with 0.5% Triton X-100 in PBST and then 20 mins in 3% H_2_O_2_, a step required for signal amplification. After that, embryos were incubated for 2 h in a blocking solution (PBST with 3% BSA and 5% NGS) and then with a primary antibody. For signal amplification, embryos were incubated with HRP-conjugated goat anti-rabbit and then in a staining buffer containing Alexa Fluor 647 (Tyramide SuperBoost Kit, Thermo Fisher Scientific). In Figs 1G-J, 2E-F′ and 3A,B, nuclei were visualized using DAPI (4,6-diamidino-2-phenylindole; Sigma-Aldrich) (1:1000) staining. To image the cardiac cone at 20 hpf and the heart tube during leftward jogging, embryos were manually deyolked, flattened and mounted in SlowFade Gold (Thermo Fisher Scientific) for imaging. All 48 hpf embryonic hearts were manually extracted and mounted in SlowFade Gold. Images were recorded on an LSM 880 confocal microscope (Zeiss) and processed with Fiji in order to minimize the background and highlight the signal. For each experiment, the same imaging settings were used. Fiji software was also used to obtain the *x*-*z* orthogonal view of the cardiac cone at 20 hpf as shown in Fig. 4A″,B″.

### Live imaging

Live imaging was performed using a light-sheet Z.1 microscope (Carl Zeiss) equipped with a water immersion 20× detection objective lens (W Plan Apochromat, NA 1.0), dual-sided 10× illumination objective lenses (LSMF, NA 0.2), a pco.edge scientific CMOS camera (PCO) and ZEN software. Embryos were manually dechorionated, anesthetized in 0.16 mg/ml Tricaine in egg water (60 µg/ml sea salt) (this solution was also used to fill the light-sheet chamber during imaging), transferred into 1% low-melting agarose (Lonza 50081) containing 0.16 mg/ml Tricaine in egg water and withdrawn into glass capillaries using a metal plunger. Embryos were positioned with the dorsal side facing the outer wall of the agarose cylinder. For the time-lapse datasets of the heart field during leftward jogging (Movies 1, 2), *z*-stacks encompassing the entire heart field (based on GFP fluorescence), a *z*-interval of 1 µm, exposure of 30-50 msec, and a time interval of 5 min for 100 cycles for wild-type and 10 min for 100 cycles for *wnt9^DKO^* were used. For the time-lapse datasets of the heart field during cardiomyocyte progenitor cell migrations towards the embryonic midline (Movies 4, 5), *z*-stacks encompassing the entire heart field (based on GFP fluorescence), a *z*-interval of 1 µm, exposure of 30-50 msec and a time interval of 2.5 min for 120 cycles were used.

### Imaging analysis

The tracking spot function of Imaris software (Oxford Instruments) was used for cell tracking analyses shown in Fig. 2A-D. The estimated diameter of each object/cell was set at 7.5 µm. Only cardiomyocyte progenitor cells in anterior positions, which were visible for at least five consecutive time points, were considered for analyses. These cells were highlighted by light red dots in wild-type (Fig. 2A; Movie 4) and cyan dots in *wnt9a*/*b* double morphants. (Fig. 2B; Movie 5). The cardiomyocyte migration straightness index and velocity were determined by the same software and tracking algorithm. A straightness index equal to 1 indicates a cell that migrates straight over some distance, whereas a value of 0 indicates that the cell trembles and oscillates around the same position. Each dot in Fig. 2C,D represents the average of the straightness index (Fig. 2C) and velocity of cardiomyocytes (Fig. 2D) for each embryo analyzed. The averages of the straightness index and velocity between wild-type and *wnt9a*/*b* double morphants were compared and the statistical significance of the resulting difference was determined by a two-tailed, unpaired Student's *t*-test, using GraphPad Prism (version 9). The average values of cardiomyocyte progenitor cell straightness index of migration and velocity are indicated in Table 3.

**Table 3.**
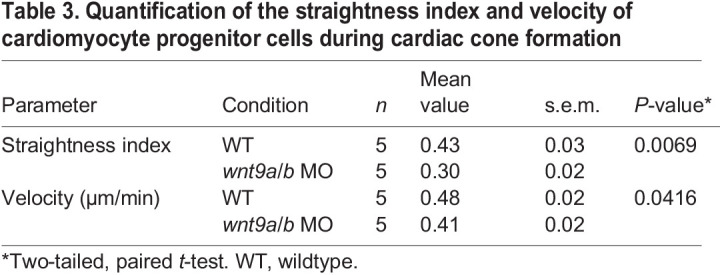
Quantification of the straightness index and velocity of cardiomyocyte progenitor cells during cardiac cone formation

Imaris software (Oxford Instruments; counting spots function) was used for counting cardiomyocytes as shown in Fig. 3A-C. To better distinguish cardiomyocytes for automatic counting, cardiomyocyte-specific EGFP was colocalized with DAPI. The automatic counting was done only with nuclei that were EGFP/DAPI double positive. The averages of the cardiomyocyte numbers between wild-type and *wnt9^DKO^* mutants were compared and the statistical significance of the resulting difference was determined by a two-tailed, unpaired Student's *t*-test, using GraphPad Prism (version 9). The average values of cardiomyocyte numbers are indicated in Table 4.

**Table 4.**
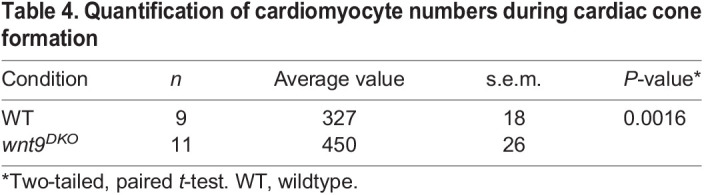
Quantification of cardiomyocyte numbers during cardiac cone formation

**Table 5:**
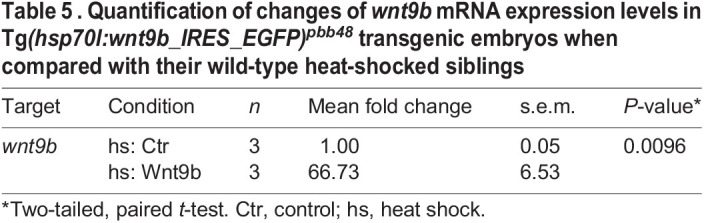
. Quantification of changes of *wnt9b* mRNA expression levels in Tg*(hsp70l:wnt9b_IRES_EGFP)^pbb48^* transgenic embryos when compared with their wild-type heat-shocked siblings

### Quantification and statistical analysis

All statistical analyses were performed with GraphPad Prism (version 9). Data representations and *P*-value calculations are indicated in the figure legends and method details. All indicated *P*-values are two-tailed and significance was defined as *P*<0.05.

## Supplementary Material

10.1242/develop.201707_sup1 Supplementary information

## References

[DEV201707C1] Akieda, Y., Ogamino, S., Furuie, H., Ishitani, S., Akiyoshi, R., Nogami, J., Masuda, T., Shimizu, N., Ohkawa, Y. and Ishitani, T. (2019). Cell competition corrects noisy Wnt morphogen gradients to achieve robust patterning in the zebrafish embryo. *Nat. Commun.* 10, 4710. 10.1038/s41467-019-12609-4 31624259 PMC6797755

[DEV201707C2] Bakkers, J. (2011). Zebrafish as a model to study cardiac development and human cardiac disease. *Cardiovasc. Res.* 91, 279-288. 10.1093/cvr/cvr098 21602174 PMC3125074

[DEV201707C3] Beis, D., Bartman, T., Jin, S. W., Scott, I. C., D'amico, L. A., Ober, E. A., Verkade, H., Frantsve, J., Field, H. A., Wehman, A. et al. (2005). Genetic and cellular analyses of zebrafish atrioventricular cushion and valve development. *Development* 132, 4193-4204. 10.1242/dev.01970 16107477

[DEV201707C4] Bussmann, J., Bakkers, J. and Schulte-Merker, S. (2007). Early endocardial morphogenesis requires Scl/Tal1. *PLoS Genet.* 3, e140. 10.1371/journal.pgen.0030140 17722983 PMC1950955

[DEV201707C5] Carroll, T. J., Park, J.-S., Hayashi, S., Majumdar, A. and Mcmahon, A. P. (2005). Wnt9b plays a central role in the regulation of mesenchymal to epithelial transitions underlying organogenesis of the mammalian urogenital system. *Dev. Cell* 9, 283-292. 10.1016/j.devcel.2005.05.016 16054034

[DEV201707C6] Carvalho, J. R., Fortunato, I. C., Fonseca, C. G., Pezzarossa, A., Barbacena, P., Dominguez-Cejudo, M. A., Vasconcelos, F. F., Santos, N. C., Carvalho, F. A. and Franco, C. A. (2019). Non-canonical Wnt signaling regulates junctional mechanocoupling during angiogenic collective cell migration. *eLife* 8, e45853. 10.7554/eLife.45853 31246175 PMC6684320

[DEV201707C7] Chen, B., Dodge, M. E., Tang, W., Lu, J., Ma, Z., Fan, C.-W., Wei, S., Hao, W., Kilgore, J., Williams, N. S. et al. (2009). Small molecule–mediated disruption of Wnt-dependent signaling in tissue regeneration and cancer. *Nat. Chem. Biol.* 5, 100-107. 10.1038/nchembio.137 19125156 PMC2628455

[DEV201707C8] Choudhry, P. and Trede, N. S. (2013). DiGeorge syndrome gene tbx1 functions through wnt11r to regulate heart looping and differentiation. *PLoS One* 8, e58145. 10.1371/journal.pone.0058145 23533583 PMC3606275

[DEV201707C9] Daneman, R., Agalliu, D., Zhou, L., Kuhnert, F., Kuo, C. J. and Barres, B. A. (2009). Wnt/beta-catenin signaling is required for CNS, but not non-CNS, angiogenesis. *Proc. Natl. Acad. Sci. USA* 106, 641-646. 10.1073/pnas.0805165106 19129494 PMC2626756

[DEV201707C10] Denning, C., Borgdorff, V., Crutchley, J., Firth, K. S. A., George, V., Kalra, S., Kondrashov, A., Hoang, M. D., Mosqueira, D., Patel, A. et al. (2016). Cardiomyocytes from human pluripotent stem cells: from laboratory curiosity to industrial biomedical platform. *Biochim. Biophys. Acta* 1863, 1728-1748. 10.1016/j.bbamcr.2015.10.014 26524115 PMC5221745

[DEV201707C11] Dickmeis, T., Mourrain, P., Saint-Etienne, L., Fischer, N., Aanstad, P., Clark, M., Strähle, U. and Rosa, F. (2001). A crucial component of the endoderm formation pathway, CASANOVA, is encoded by a novel sox-related gene. *Genes Dev.* 15, 1487-1492. 10.1101/gad.196901 11410529 PMC312720

[DEV201707C12] Dohn, T. E. and Waxman, J. S. (2012). Distinct phases of Wnt/β-catenin signaling direct cardiomyocyte formation in zebrafish. *Dev. Biol.* 361, 364-376. 10.1016/j.ydbio.2011.10.032 22094017 PMC3246556

[DEV201707C13] Field, H. A., Dong, P. D. S., Beis, D. and Stainier, D. Y. R. (2003). Formation of the digestive system in zebrafish. ii. pancreas morphogenesis⋆. *Dev. Biol.* 261, 197-208. 10.1016/S0012-1606(03)00308-7 12941629

[DEV201707C14] Fontana, F., Haack, T., Reichenbach, M., Knaus, P., Puceat, M. and Abdelilah-Seyfried, S. (2020). Antagonistic activities of Vegfr3/Flt4 and Notch1b fine-tune mechanosensitive signaling during zebrafish cardiac valvulogenesis. *Cell Rep.* 32, 107883. 10.1016/j.celrep.2020.107883 32668254

[DEV201707C15] Fukui, H., Terai, K., Nakajima, H., Chiba, A., Fukuhara, S. and Mochizuki, N. (2014). S1P-Yap1 signaling regulates endoderm formation required for cardiac precursor cell migration in zebrafish. *Dev. Cell* 31, 128-136. 10.1016/j.devcel.2014.08.014 25313964

[DEV201707C16] Gilmour, D., Rembold, M. and Leptin, M. (2017). From morphogen to morphogenesis and back. *Nature* 541, 311-320. 10.1038/nature21348 28102269

[DEV201707C17] Glickman Holtzman, N., Schoenebeck, J. J., Tsai, H. J. and Yelon, D. (2007). Endocardium is necessary for cardiomyocyte movement during heart tube assembly. *Development* 134, 2379-2386. 10.1242/dev.02857 17537802

[DEV201707C18] Goddard, L. M., Duchemin, A. L., Ramalingan, H., Wu, B., Chen, M., Bamezai, S., Yang, J., Li, L., Morley, M. P., Wang, T. et al. (2017). Hemodynamic forces sculpt developing heart valves through a KLF2-WNT9B paracrine signaling axis. *Dev. Cell* 43, 274-289.e5. 10.1016/j.devcel.2017.09.023 29056552 PMC5760194

[DEV201707C19] Grainger, S., Richter, J., Palazón, R. E., Pouget, C., Lonquich, B., Wirth, S., Grassme, K. S., Herzog, W., Swift, M. R., Weinstein, B. M. et al. (2016). Wnt9a is required for the aortic amplification of nascent hematopoietic stem cells. *Cell Rep.* 17, 1595-1606. 10.1016/j.celrep.2016.10.027 27806298 PMC6309681

[DEV201707C20] Gray, R. S., Roszko, I. and Solnica-Krezel, L. (2011). Planar cell polarity: coordinating morphogenetic cell behaviors with embryonic polarity. *Dev. Cell* 21, 120-133. 10.1016/j.devcel.2011.06.011 21763613 PMC3166557

[DEV201707C21] Horne-Badovinac, S., Lin, D., Waldron, S., Schwarz, M., Mbamalu, G., Pawson, T., Jan, Y., Stainier, D. Y. and Abdelilah-Seyfried, S. (2001). Positional cloning of heart and soul reveals multiple roles for PKC lambda in zebrafish organogenesis. *Curr. Biol.* 11, 1492-1502. 10.1016/S0960-9822(01)00458-4 11591316

[DEV201707C22] Huang, C.-J., Tu, C.-T., Hsiao, C.-D., Hsieh, F.-J. and Tsai, H.-J. (2003). Germ-line transmission of a myocardium-specific GFP transgene reveals critical regulatory elements in the cardiac myosin light chain 2 promoter of zebrafish. *Dev. Dyn.* 228, 30-40. 10.1002/dvdy.10356 12950077

[DEV201707C23] Itou, J., Oishi, I., Kawakami, H., Glass, T. J., Richter, J., Johnson, A., Lund, T. C. and Kawakami, Y. (2012). Migration of cardiomyocytes is essential for heart regeneration in zebrafish. *Development* 139, 4133-4142. 10.1242/dev.079756 23034636

[DEV201707C24] Jackson, H. W., Prakash, D., Litaker, M., Ferreira, T. and Jezewski, P. A. (2015). Zebrafish Wnt9b patterns the first pharyngeal arch into D-I-V domains and promotes anterior-medial outgrowth. *Am. J. Mol. Biol.* 05, 57-83. 10.4236/ajmb.2015.53006

[DEV201707C25] Jowett, T. and Lettice, L. (1994). Whole-mount in situ hybridizationon zebrafish embryos using a mixture of digoxigenin- and fluorescein-labelled probes. *Trends Genet.* 10, 73-74. 10.1016/0168-9525(94)90220-8 8178366

[DEV201707C26] Karner, C. M., Chirumamilla, R., Aoki, S., Igarashi, P., Wallingford, J. B. and Carroll, T. J. (2009). Wnt9b signaling regulates planar cell polarity and kidney tubule morphogenesis. *Nat. Genet.* 41, 793-799. 10.1038/ng.400 19543268 PMC2761080

[DEV201707C27] Kikuchi, K., Holdway, J. E., Werdich, A. A., Anderson, R. M., Fang, Y., Egnaczyk, G. F., Evans, T., Macrae, C. A., Stainier, D. Y. R. and Poss, K. D. (2010). Primary contribution to zebrafish heart regeneration by gata4+ cardiomyocytes. *Nature* 464, 601-605. 10.1038/nature08804 20336144 PMC3040215

[DEV201707C28] Komiya, Y. and Habas, R. (2008). Wnt signal transduction pathways. *Organogenesis* 4, 68-75. 10.4161/org.4.2.5851 19279717 PMC2634250

[DEV201707C29] Livak, K. J. and Schmittgen, T. D. (2001). Analysis of relative gene expression data using real-time quantitative PCR and the 2-ΔΔCT method. *Methods* 25, 402-408. 10.1006/meth.2001.1262 11846609

[DEV201707C30] Long, S., Ahmad, N. and Rebagliati, M. (2003). The zebrafish nodal-related gene southpaw is required for visceral and diencephalic left-right asymmetry. *Development* 130, 2303-2316. 10.1242/dev.00436 12702646

[DEV201707C31] Lu, J., Ma, Z., Hsieh, J.-C., Fan, C.-W., Chen, B., Longgood, J. C., Williams, N. S., Amatruda, J. F., Lum, L. and Chen, C. (2009). Structure-activity relationship studies of small-molecule inhibitors of Wnt response. *Bioorg. Med. Chem. Lett.* 19, 3825-3827. 10.1016/j.bmcl.2009.04.040 19410457 PMC2709695

[DEV201707C32] Matsui, T., Raya, A., Kawakami, Y., Callol-Massot, C., Capdevila, J., Rodríguez-Esteban, C. and Izpisúa Belmonte, J. C. (2005). Noncanonical Wnt signaling regulates midline convergence of organ primordia during zebrafish development. *Genes Dev.* 19, 164-175. 10.1101/gad.1253605 15630025 PMC540234

[DEV201707C33] Merks, A. M., Swinarski, M., Meyer, A. M., Müller, N. V., Özcan, I., Donat, S., Burger, A., Gilbert, S., Mosimann, C., Abdelilah-Seyfried, S. et al. (2018). Planar cell polarity signalling coordinates heart tube remodelling through tissue-scale polarisation of actomyosin activity. *Nat. Commun.* 9, 2161. 10.1038/s41467-018-04566-1 29867082 PMC5986786

[DEV201707C34] Montcouquiol, M., Crenshaw, E. B. and Kelley, M. W. (2006). Noncanonical WNT signaling and neural polarity. *Annu. Rev. Neurosci.* 29, 363-386. 10.1146/annurev.neuro.29.051605.112933 16776590

[DEV201707C35] Moro, E., Ozhan-Kizil, G., Mongera, A., Beis, D., Wierzbicki, C., Young, R. M., Bournele, D., Domenichini, A., Valdivia, L. E., Lum, L. et al. (2012). In vivo Wnt signaling tracing through a transgenic biosensor fish reveals novel activity domains. *Dev. Biol.* 366, 327-340. 10.1016/j.ydbio.2012.03.023 22546689

[DEV201707C36] Murashov, A. K., Pak, E. S. and Katwa, L. C. (2005). Parallel development of cardiomyocytes and neurons in embryonic stem cell culture. *Biochem. Biophys. Res. Commun.* 332, 653-656. 10.1016/j.bbrc.2005.04.167 15894285

[DEV201707C37] Nicoli, S., Standley, C., Walker, P., Hurlstone, A., Fogarty, K. E. and Lawson, N. D. (2010). MicroRNA-mediated integration of haemodynamics and Vegf signalling during angiogenesis. *Nature* 464, 1196-1200. 10.1038/nature08889 20364122 PMC2914488

[DEV201707C38] Paaby, A. B. and Rockman, M. V. (2013). The many faces of pleiotropy. *Trends Genet.* 29, 66-73. 10.1016/j.tig.2012.10.010 23140989 PMC3558540

[DEV201707C39] Paolini, A., Fontana, F., Pham, V. C., Rödel, C. J. and Abdelilah-Seyfried, S. (2021). Mechanosensitive Notch-Dll4 and Klf2-Wnt9 signaling pathways intersect in guiding valvulogenesis in zebrafish. *Cell Rep*. 37, 109782. 10.1016/j.celrep.2021.109782 34610316 PMC8511505

[DEV201707C40] Paredes, I., Himmels, P. and Ruiz De Almodóvar, C. (2018). Neurovascular communication during CNS development. *Dev. Cell* 45, 10-32. 10.1016/j.devcel.2018.01.023 29634931

[DEV201707C41] Park, H.-C., Kim, C.-H., Bae, Y.-K., Yeo, S.-Y., Kim, S.-H., Hong, S.-K., Shin, J., Yoo, K.-W., Hibi, M., Hirano, T. et al. (2000). Analysis of upstream elements in the HuC promoter leads to the establishment of transgenic zebrafish with fluorescent neurons. *Dev. Biol.* 227, 279-293. 10.1006/dbio.2000.9898 11071755

[DEV201707C42] Peguera, B., Segarra, M. and Acker-Palmer, A. (2021). Neurovascular crosstalk coordinates the central nervous system development. *Curr. Opin. Neurobiol.* 69, 202-213. 10.1016/j.conb.2021.04.005 34077852 PMC8411665

[DEV201707C43] Proulx, K., Lu, A. and Sumanas, S. (2010). Cranial vasculature in zebrafish forms by angioblast cluster-derived angiogenesis. *Dev. Biol.* 348, 34-46. 10.1016/j.ydbio.2010.08.036 20832394

[DEV201707C44] Reischauer, S., Stone, O. A., Villasenor, A., Chi, N., Jin, S. W., Martin, M., Lee, M. T., Fukuda, N., Marass, M., Witty, A. et al. (2016). Cloche is a bHLH-PAS transcription factor that drives haemato-vascular specification. *Nature* 535, 294-298. 10.1038/nature18614 27411634

[DEV201707C45] Renz, M., Otten, C., Faurobert, E., Rudolph, F., Zhu, Y., Boulday, G., Duchene, J., Mickoleit, M., Dietrich, A. C., Ramspacher, C. et al. (2015). Regulation of β1 integrin-Klf2-mediated angiogenesis by CCM proteins. *Dev. Cell* 32, 181-190. 10.1016/j.devcel.2014.12.016 25625207

[DEV201707C46] Rohr, S., Bit-Avragim, N. and Abdelilah-Seyfried, S. (2006). Heart and soul/PRKCi and nagie oko/Mpp5 regulate myocardial coherence and remodeling during cardiac morphogenesis. *Development* 133, 107-115. 10.1242/dev.02182 16319113

[DEV201707C47] Rohr, S., Otten, C. and Abdelilah-Seyfried, S. (2008). Asymmetric involution of the myocardial field drives heart tube formation in zebrafish. *Circ. Res.* 102, 12-19. 10.1161/CIRCRESAHA.107.165241 18202314

[DEV201707C48] Ruiz-Villalba, A., Hoppler, S. and Van Den Hoff, M. J. B. (2016). Wnt signaling in the heart fields: variations on a common theme. *Dev. Dyn.* 245, 294-306. 10.1002/dvdy.24372 26638115

[DEV201707C49] Santos-Ledo, A., Washer, S., Dhanaseelan, T., Eley, L., Alqatani, A., Chrystal, P. W., Papoutsi, T., Henderson, D. J. and Chaudhry, B. (2020). Alternative splicing of jnk1a in zebrafish determines first heart field ventricular cardiomyocyte numbers through modulation of hand2 expression. *PLoS Genet.* 16, e1008782. 10.1371/journal.pgen.1008782 32421721 PMC7259801

[DEV201707C50] Schindelin, J., Arganda-Carreras, I., Frise, E., Kaynig, V., Longair, M., Pietzsch, T., Preibisch, S., Rueden, C., Saalfeld, S., Schmid, B. et al. (2012). Fiji: an open-source platform for biological-image analysis. *Nat. Methods* 9, 676-682. 10.1038/nmeth.2019 22743772 PMC3855844

[DEV201707C51] Smith, K. A., Chocron, S., Von Der Hardt, S., De Pater, E., Soufan, A., Bussmann, J., Schulte-Merker, S., Hammerschmidt, M. and Bakkers, J. (2008). Rotation and asymmetric development of the zebrafish heart requires directed migration of cardiac progenitor cells. *Dev. Cell* 14, 287-297. 10.1016/j.devcel.2007.11.015 18267096

[DEV201707C52] Trinh, L. A. and Stainier, D. Y. R. (2004). Fibronectin regulates epithelial organization during myocardial migration in zebrafish. *Dev. Cell* 6, 371-382. 10.1016/S1534-5807(04)00063-2 15030760

[DEV201707C53] Ueno, S., Weidinger, G., Osugi, T., Kohn, A. D., Golob, J. L., Pabon, L., Reinecke, H., Moon, R. T. and Murry, C. E. (2007). Biphasic role for Wnt/β-catenin signaling in cardiac specification in zebrafish and embryonic stem cells. *Proc. Natl. Acad. Sci. USA* 104, 9685-9690. 10.1073/pnas.0702859104 17522258 PMC1876428

[DEV201707C54] Van Amerongen, R. and Nusse, R. (2009). Towards an integrated view of Wnt signaling in development. *Development* 136, 3205-3214. 10.1242/dev.033910 19736321

[DEV201707C55] Vincent, J.-P. and Briscoe, J. (2001). Morphogens. *Curr. Biol.* 11, R851-R854. 10.1016/S0960-9822(01)00514-0 11696339

[DEV201707C56] Westerfield, M., Doerry, E., Kirkpatrick, A. E., Driever, W. and Douglas, S. A. (1997). An on-line database for zebrafish development and genetics research. *Semin. Cell Dev. Biol.* 8, 477-488. 10.1006/scdb.1997.0173 9441953

[DEV201707C57] Witzel, H. R., Jungblut, B., Choe, C. P., Crump, J. G., Braun, T. and Dobreva, G. (2012). The LIM protein Ajuba restricts the second heart field progenitor pool by regulating Isl1 activity. *Dev. Cell* 23, 58-70. 10.1016/j.devcel.2012.06.005 22771034 PMC3671491

[DEV201707C58] Yelon, D., Horne, S. A. and Stainier, D. Y. (1999). Restricted expression of cardiac myosin genes reveals regulated aspects of heart tube assembly in zebrafish. *Dev. Biol.* 214, 23-37. 10.1006/dbio.1999.9406 10491254

[DEV201707C59] Zhang, Y., Yeh, J. R., Mara, A., Ju, R., Hines, J. F., Cirone, P., Griesbach, H. L., Schneider, I., Slusarski, D. C., Holley, S. A. et al. (2006). A chemical and genetic approach to the mode of action of fumagillin. *Chem. Biol.* 13, 1001-1009. 10.1016/j.chembiol.2006.07.010 16984890 PMC2583369

