# Correction: Wnt9 directs zebrafish heart tube assembly via a combination of canonical and non-canonical pathway signaling

**DOI:** 10.1242/dev.202439

**Published:** 2023-11-06

**Authors:** Alessio Paolini, Dinara Sharipova, Tim Lange, Salim Abdelilah-Seyfried

There were errors in *Development* (2023) **150**, dev201707 (doi:10.1242/dev.201707).

Outdated gene symbols were used for *wnt11f1* and *wnt11f2*.

**Corrected:**

*wnt11f2* (previously known as *wnt11*/*silberblick*) and *wnt5b* trigger PCP signaling during cardiac looping […]

**Original:**

*wnt11* and *wnt5b* trigger PCP signaling during cardiac looping […]

**Corrected:**

In *wnt11f2* (Matsui et al., 2005) or *wnt11f1* (previously known as *wnt11r*) (Choudhry and Trede, 2013) mutants […]

**Original:**

In *wnt11* (Matsui et al., 2005) or *wnt11r* (Choudhry and Trede, 2013) mutants […]

The authors apologise for these errors and any inconvenience they may have caused. They do not impact the results or conclusions of this article. Both the online full-text and PDF versions of the paper have been corrected.

