# Supplementary material for: Wnt9 directs zebrafish heart tube assembly via a combination of canonical and non-canonical pathway signaling
Source: Development. 2023 Sep 25;150(18):dev201707. doi: 10.1242/dev.201707 (PMC10560569; doi:10.1242/dev.201707)
Supplement: Supplementary information [file develop-150-201707-s1.pdf]

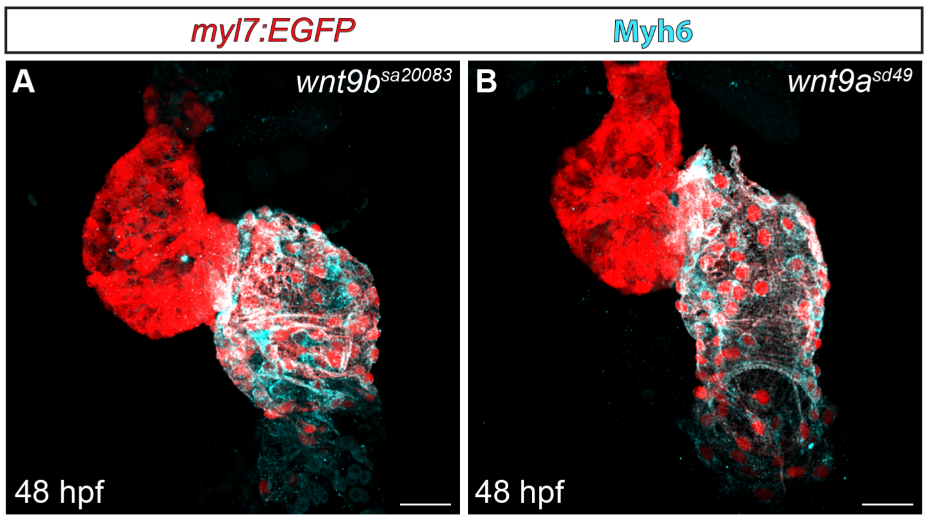

Figure S1

Fig. S1. ***wnt9b**$^{sa20083/sa20083}$* **and** ***wnt9a**$^{sd49/sd49}$* **single mutants lack early cardiac defects.** **(A-B)** Maximum projections of confocal z-scans of 48 hpf zebrafish hearts. *wnt9b*$^{sa20083/}$$_{sa20083}$ and *wnt9a*$^{sd49/sd49}$ single mutants have a correctly looped heart. Scale bars: 30 µm.

Development • Supplementary information

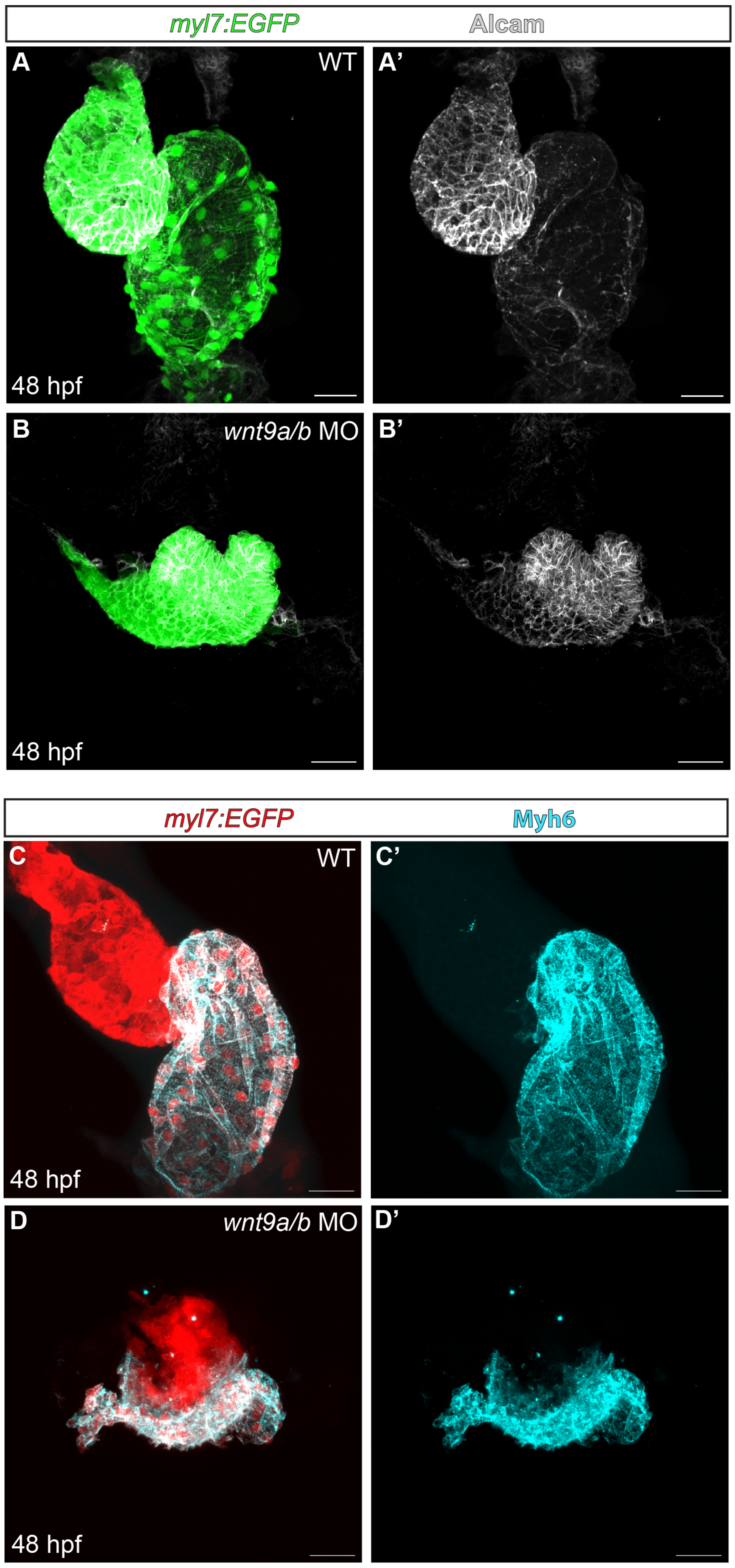

Figure S2

Fig. S2. Early cardiac phenotypes in *wnt9a/b* double morphants.

(A-D') Maximum projections of confocal z-scans of 48 hpf zebrafish hearts. (A-B') Myocardial tissue is counter-labeled with an antibody against Alcam, (C-D') while atrial cardiomyocytes are counter-labeled with Myh6. (A,A',C,C') Differently from the looped wild-type heart, (B,B',D,D') the heart collapses in *wnt9a/b* double morphants. Scale bars: 30 µm.

Development • Supplementary information

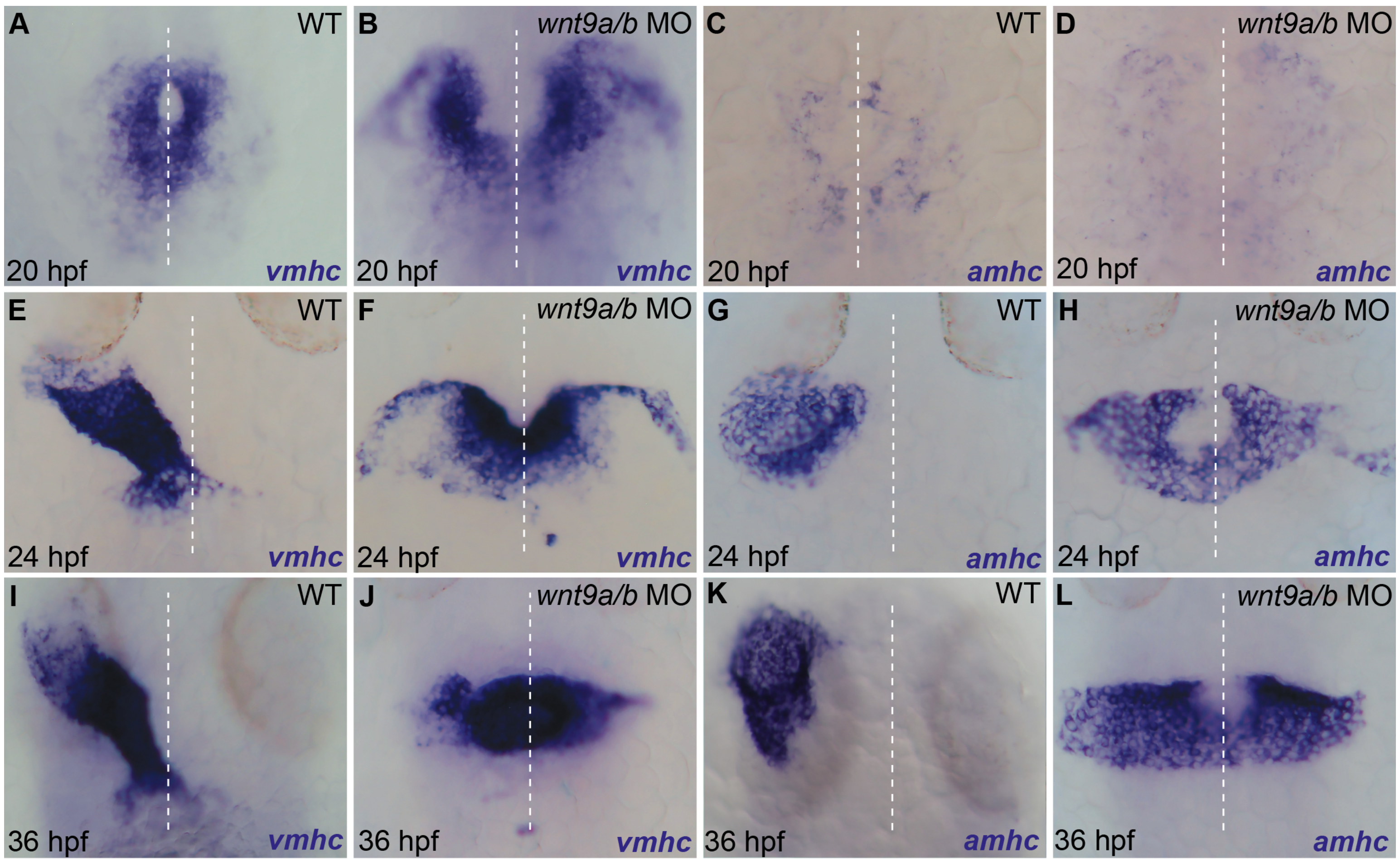

Figure S3

Fig. S3. *wnt9a/b* paralogues genes orchestrate early myocardial morphogenesis.

(A-L) Whole-mount *in situ* hybridization of *vmhc* (A,B,E,F,I,J) and *amhc* (C,D,G,H,K,L) expression during stages of cardiac cone formation and leftward jogging. (A,C) At 20 hpf, wild-type atrial and ventricular cardiomyocyte progenitor cells converge and fuse at the embryonic midline. (B,D) In *wnt9a/b* double morphants, cardiac progenitor cells do not converge and fail to fuse anteriorly. (E,G) In the 24 hpf wild-type heart, both atrial and ventricular cardiomyocyte progenitor cells undergo leftward jogging, (F,H) while in *wnt9a/b* double morphants, cardiomyocyte progenitor cells remain at the embryonic midline. (I,K) In the 36 hpf wild-type heart, the expression of *vmhc* and *amhc* marks the elongating heart tube, while (J,L) in *wnt9a/b* double morphants, cardiomyocyte progenitor cells are still located at the embryonic midline. The embryonic midline is indicated by dashed lines.

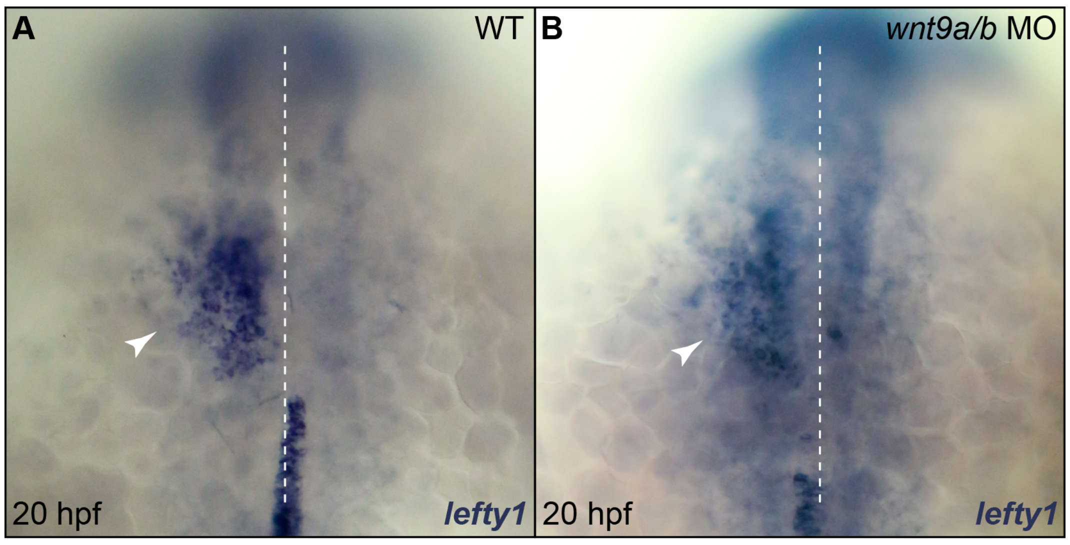

Figure S4

Fig. S4. *wnt9a/b* paralogous genes orchestrate early myocardial morphogenesis independently of left/right asymmetry signaling pathways.

(A,B) Whole-mount *in situ* hybridization of *lefty1* expression during the stages of cardiac cone formation. (A) The expression domains of *lefty1* in wild-type embryos and (B) *wnt9a/b* double morphants are unchanged. Arrowheads indicate the *lefty1* expression domain and dashed lines highlight the embryonic midline.

Development • Supplementary information

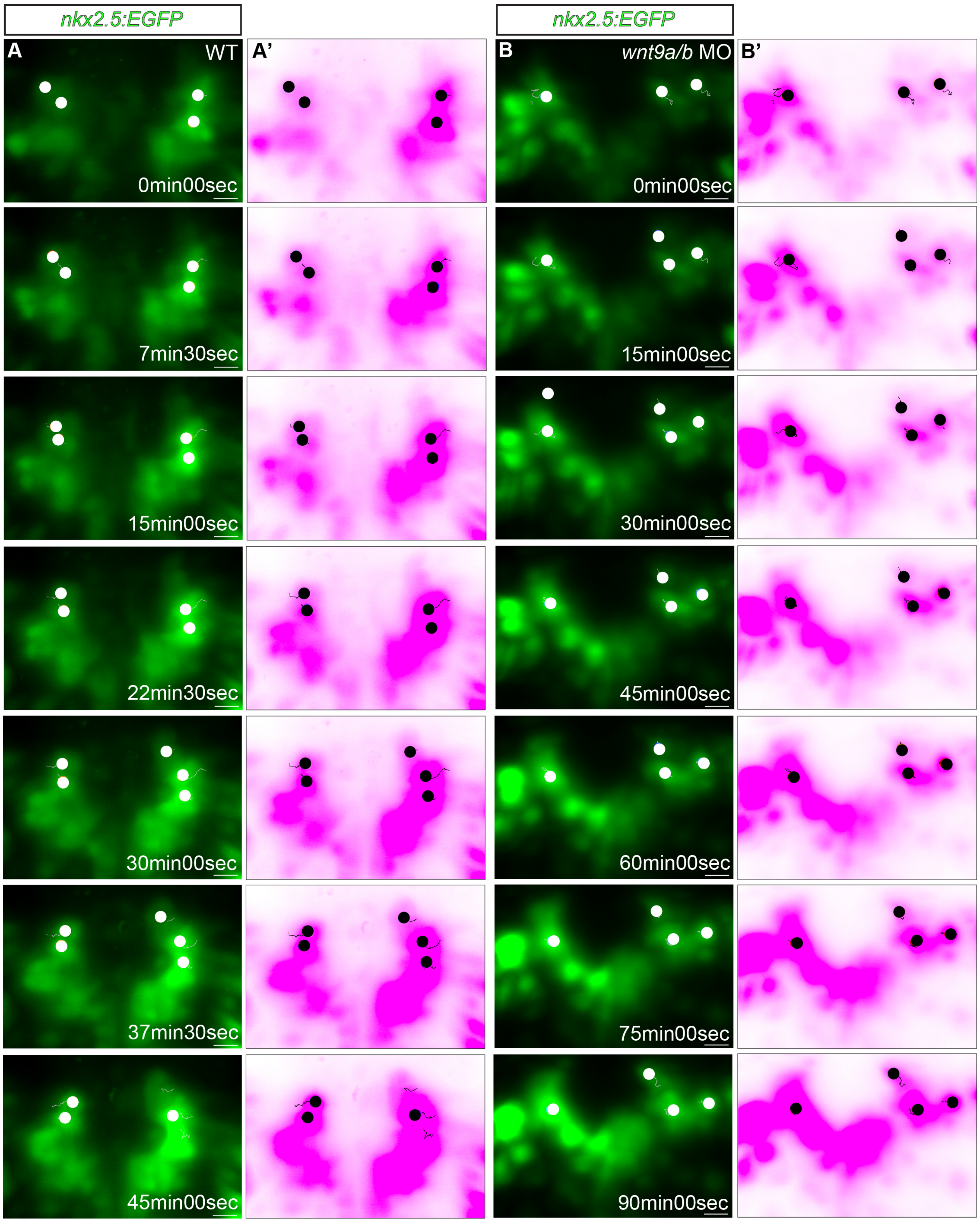

Figure S5

Fig. S5. Loss of Wnt9a/b affects cardiac progenitor cell migrations during cardiac cone formation. (A-B'). Tracking details of cardiomyocyte progenitors based on light sheet microscopy-derived time-lapse movies. (A) Anterior cardiomyocytes in wild-type embryos migrate directly towards the embryonic midline. (B) *wnt9^{DKO}* cardiomyocytes in anterior positions meander in a less directional manner. (A',B'). Shown are inverted colors to visualize cell tracks. White dots highlight individual cells. Scale bars: 20 µm

Development • Supplementary information

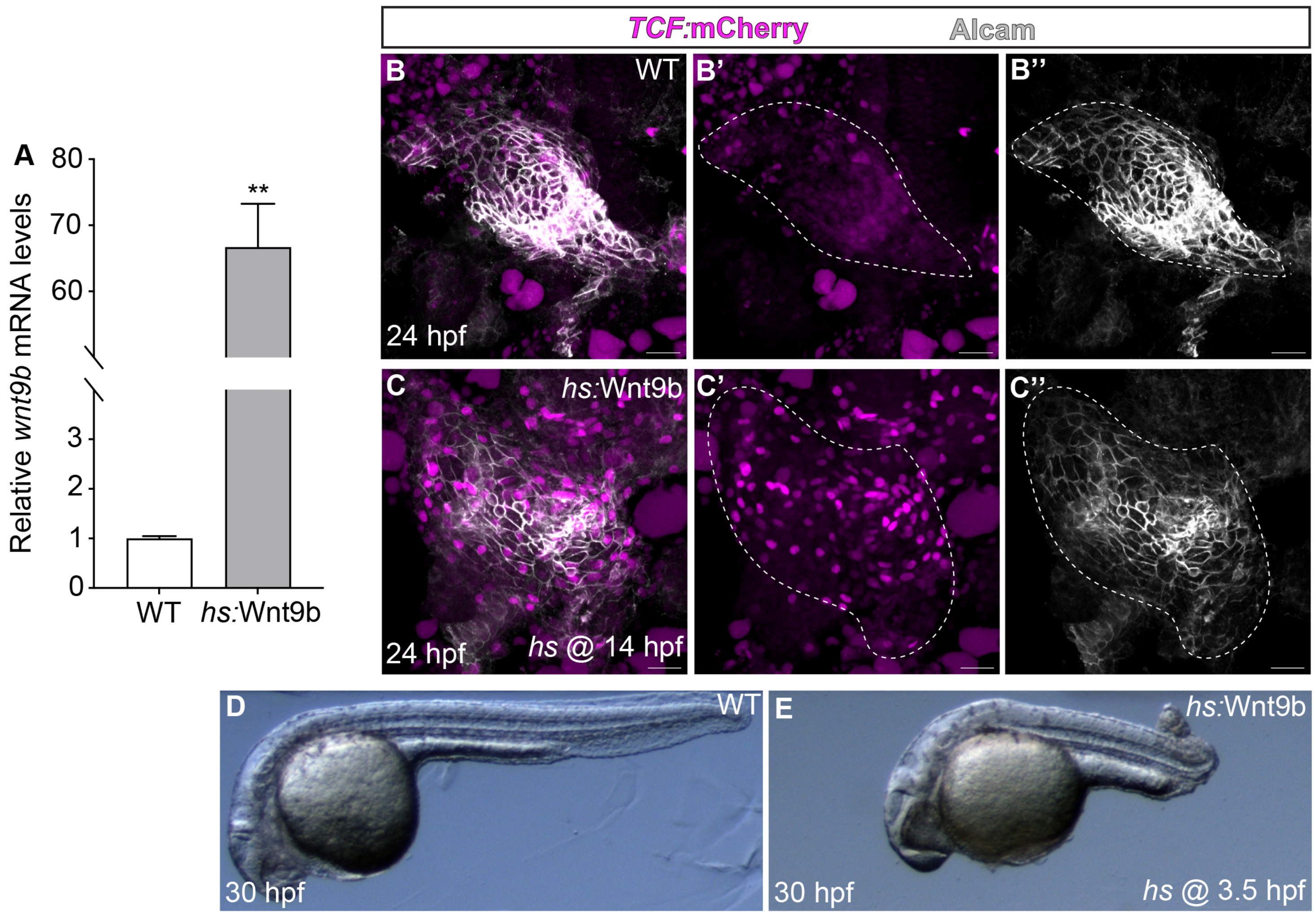

Figure S6

Fig. S6. Heat-shock of Tg*(hsp70l:wnt9b_IRES_EGFP)*^{pbb48} embryos activates canonical Wnt signaling.

(A). Quantifications of *wnt9b* mRNA expression levels by quantitative real-time PCR in Tg*(hsp70l:wnt9b_IRES_EGFP)*^{pbb48} transgenic embryos when compared with their wild-type heat-shocked siblings (n = 3 experiments; **p < 0.01 by paired Student's t-test).

(B-C). Maximum projections of confocal z-scan section planes with ventral views of the zebrafish heart field during leftward jogging at 24 hpf. (B-B") In the wild-type heart, only few cells activate the canonical Wnt reporter Tg*(7xTCF-Xla.Siam:nlsmCherry)*^{ia5}. (C-C") Upon Wnt9b overexpression at 14 hpf, the activation of Wnt signaling increases within the elongating heart tube. White dashed line highlights the elongating heart tube, counter-labeled with an antibody against Alcam.

(D-E). Brightfield overviews of zebrafish embryos at 30 hpf. Upon heat-shock at 3.5 hpf, Tg*(hsp70l:wnt9b_IRES_EGFP)*^{pbb48} embryos are dorsalized. Scale bar: 30 µm.

Development • Supplementary information

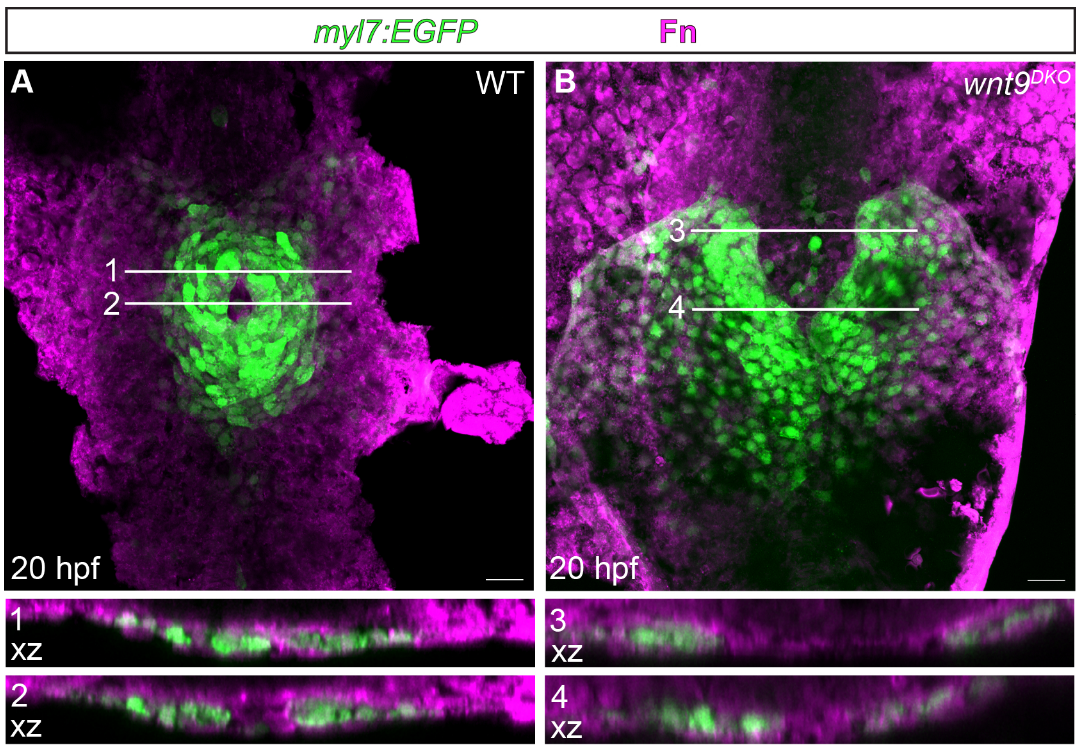

Figure S7

Fig. S7. Fn deposition is not altered in *wnt9^{DKO}*.

(A-B). Maximum projections of confocal z-scan section planes with ventral views of the zebrafish heart field during cardiac cone formation at 20 hpf. Both in WT and *wnt9^{DKO}* embryos, Fn distribution is uniform around and on the ventral side of cardiomyocyte progenitors, as shown in XZ views. Scale bar: 30 µm.

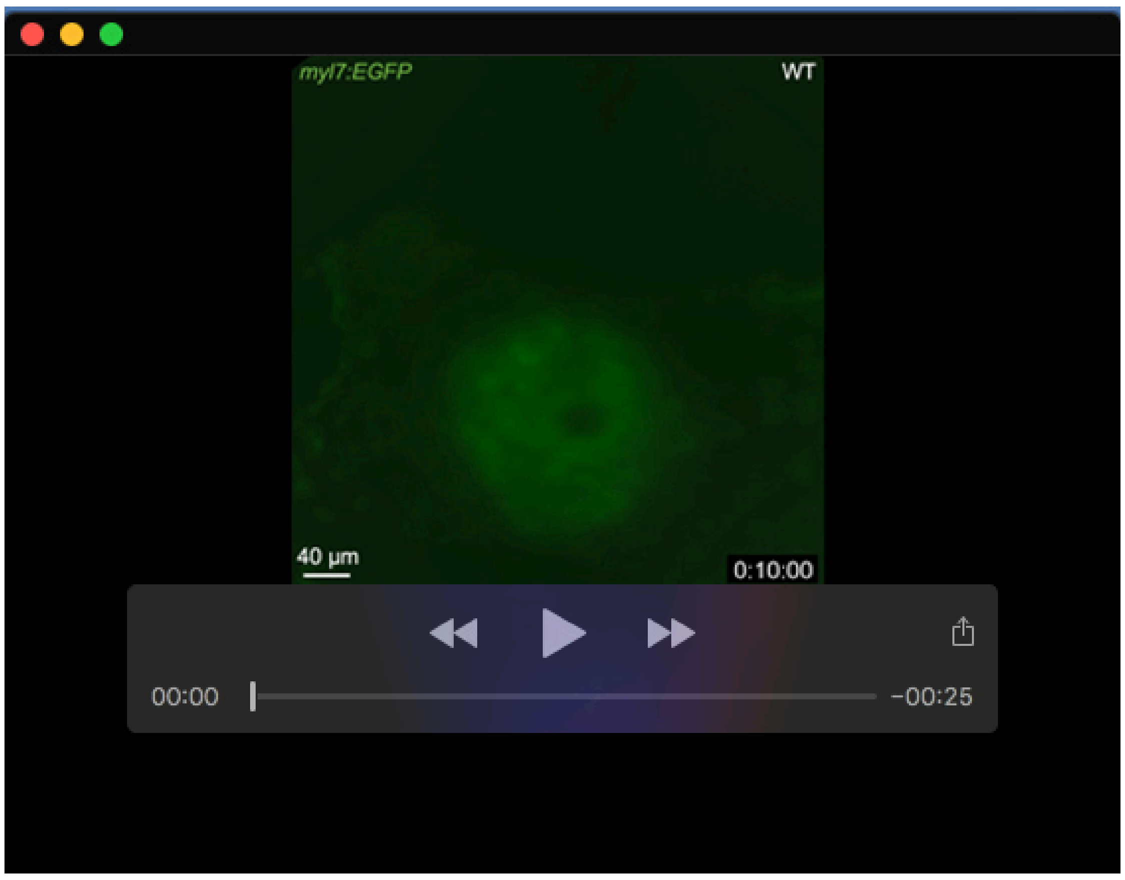

Movie 1. Leftward cardiac jogging of a wild type heart, related to Fig. 1A.

Light sheet microscopy-derived time-lapse movie of cardiomyocyte progenitor cells during leftward cardiac jogging.

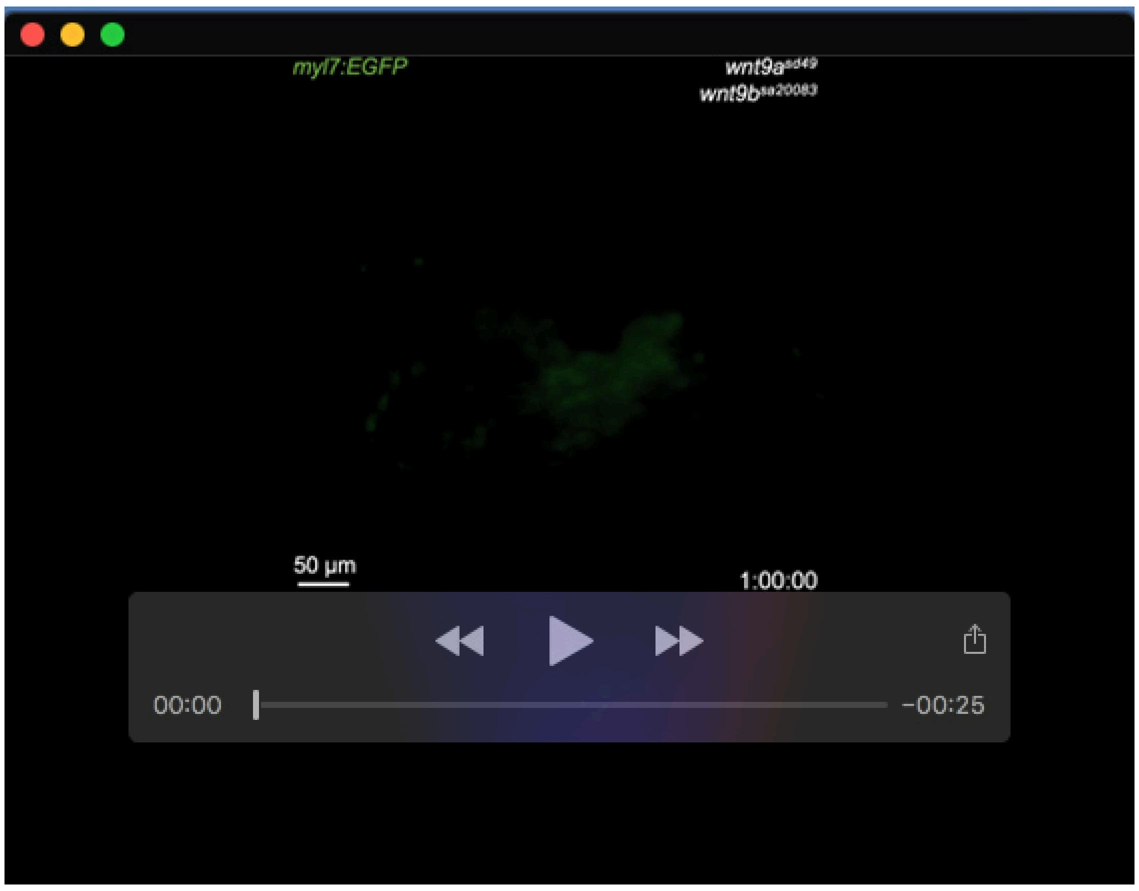

Movie 2. The *wnt9*^{DKO} heart cone fails to fuse and does not undergo leftward jogging, related to Fig. 1C.

Light sheet microscopy-derived time-lapse movie of *wnt9a/b* double morphant cardiomyocyte progenitor cells during stages of cardiac cone formation and leftward jogging.

Development • Supplementary information

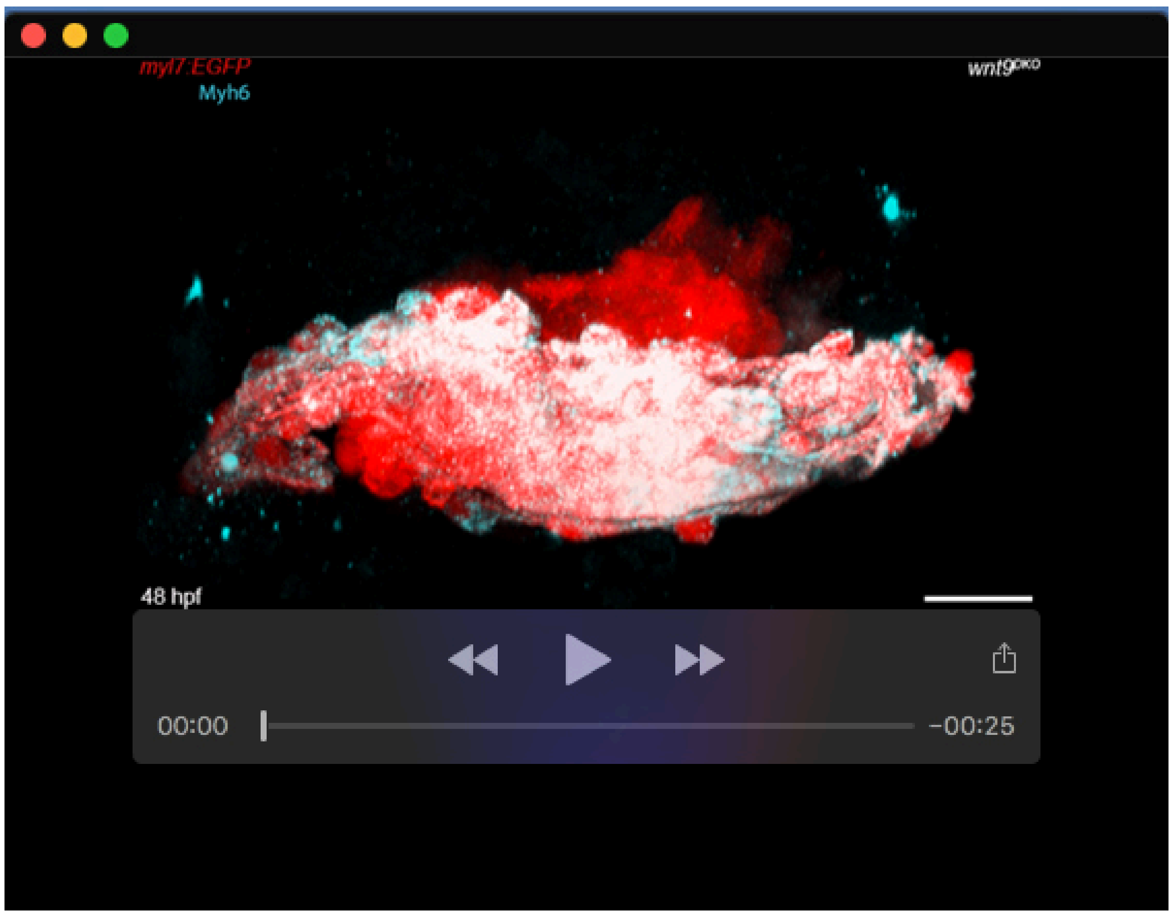

Movie 3. The *wnt9^{DKO}* mutant atrium surrounds the collapsed ventricle, related to Fig. 1C.
Shown is a X-plane-rotation of a 3D confocal reconstruction of a *wnt9^{DKO}* mutant heart at 48 hpf. The white arrow indicates where the fusion of anteriorly-positioned cardiomyocyte progenitor cells failed.

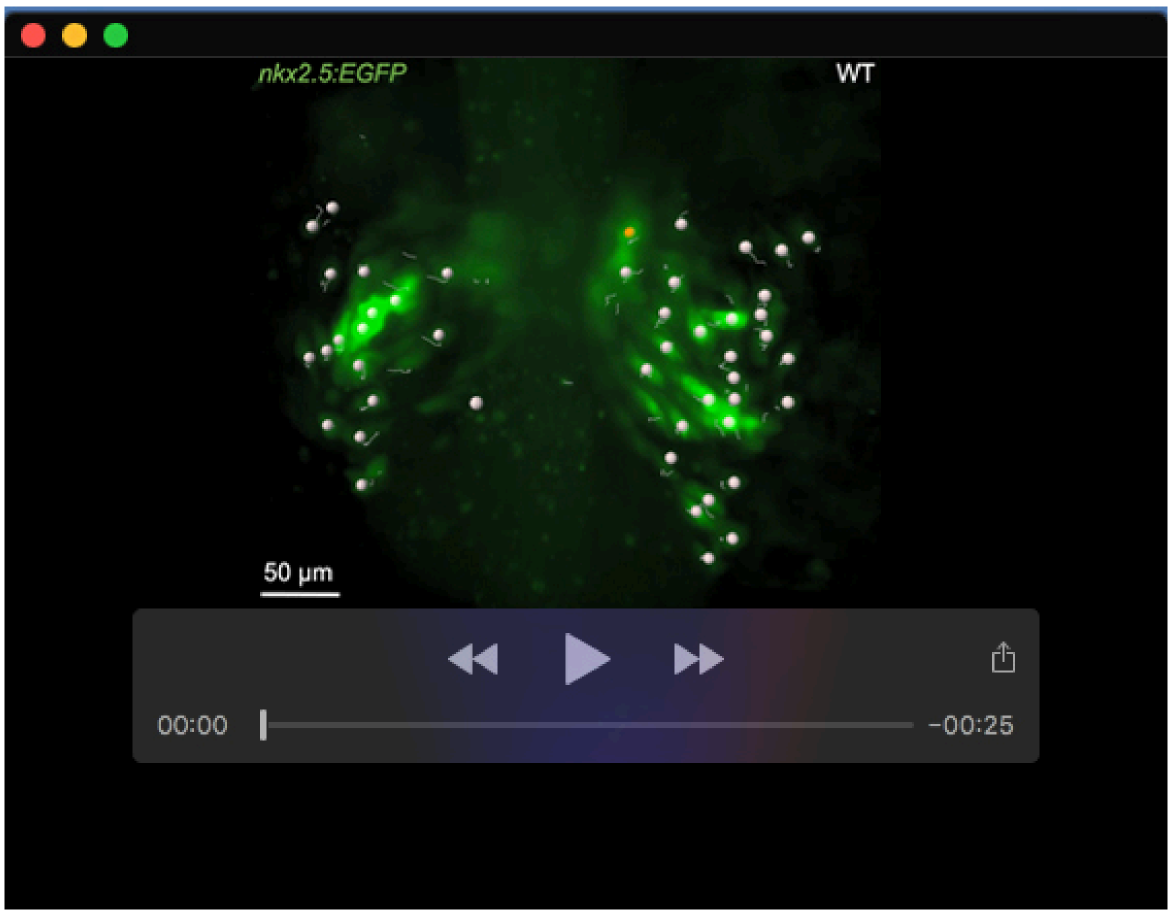

Movie 4. Tracking of cardiomyocyte progenitor cells in wild-type embryos during their migration towards the embryonic midline, related to Fig. 2A.

Light sheet microscopy-derived time-lapse movie of wild-type cardiomyocyte progenitor cells during their migration from lateral plate mesodermal positions towards the embryonic midline. Red dots indicate those cardiomyocyte progenitor cells in anterior positions that could be tracked as shown in Fig 2C,D. All other cardiomyocyte progenitor cells are marked with a white dot. Time resolution equals a frame / 150 seconds.

Development • Supplementary information

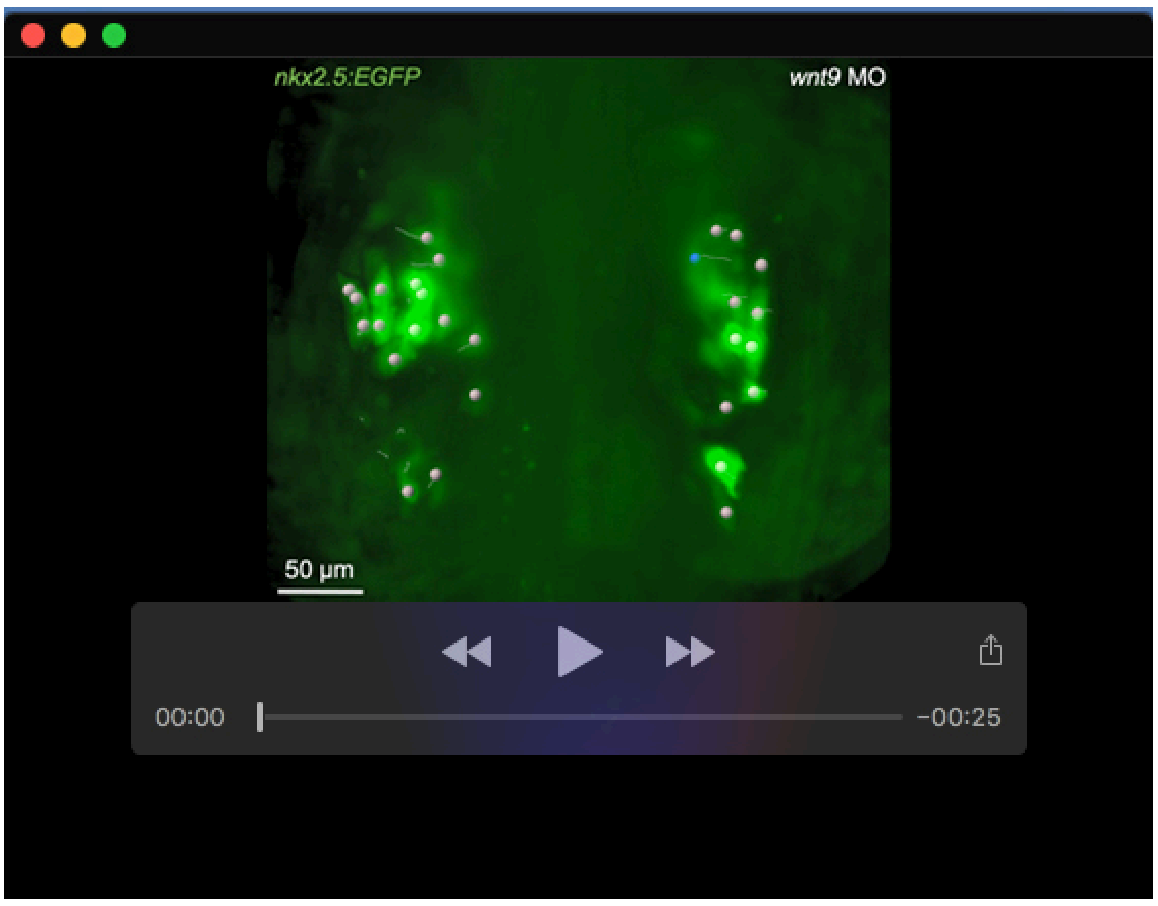

Movie 5. Tracking of cardiomyocyte progenitor cells in *wnt9^{DKO}* embryos during their migration towards the embryonic midline, related to Fig. 2B.

Light sheet microscopy-derived time-lapse movie of *wnt9^{DKO}* cardiomyocyte progenitor cells during their migration from positions in the lateral plate mesoderm towards the embryonic midline. Cyan dots indicate those cardiomyocyte progenitor cells in anterior positions that could be tracked as shown in Fig 2C,D. All other cardiomyocyte progenitor cells are marked with a white dot. Time resolution equals a frame / 150 seconds.

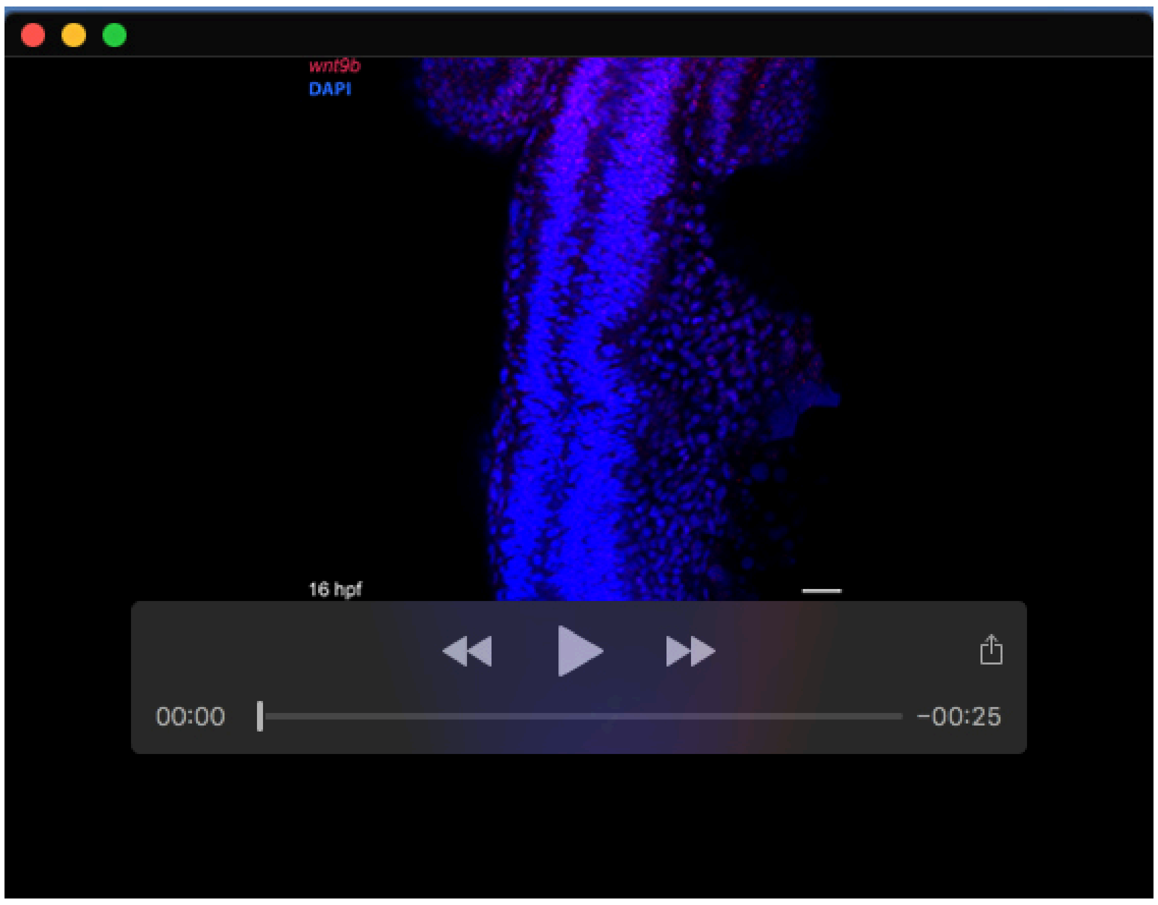

Movie 6. The lateral plate mesoderm at 16 hpf is devoid of *wnt9b* expression, related to Fig. 2E-E'.

Scroll through a series of confocal z-planes from the dorsal towards ventral side of a 16 hpf embryo. *wnt9b* expression appears stronger in ventral and anterior positions of the neural tube. Arrows point at the lateral plate mesoderm. Scale bar: 30 µm.

Development • Supplementary information

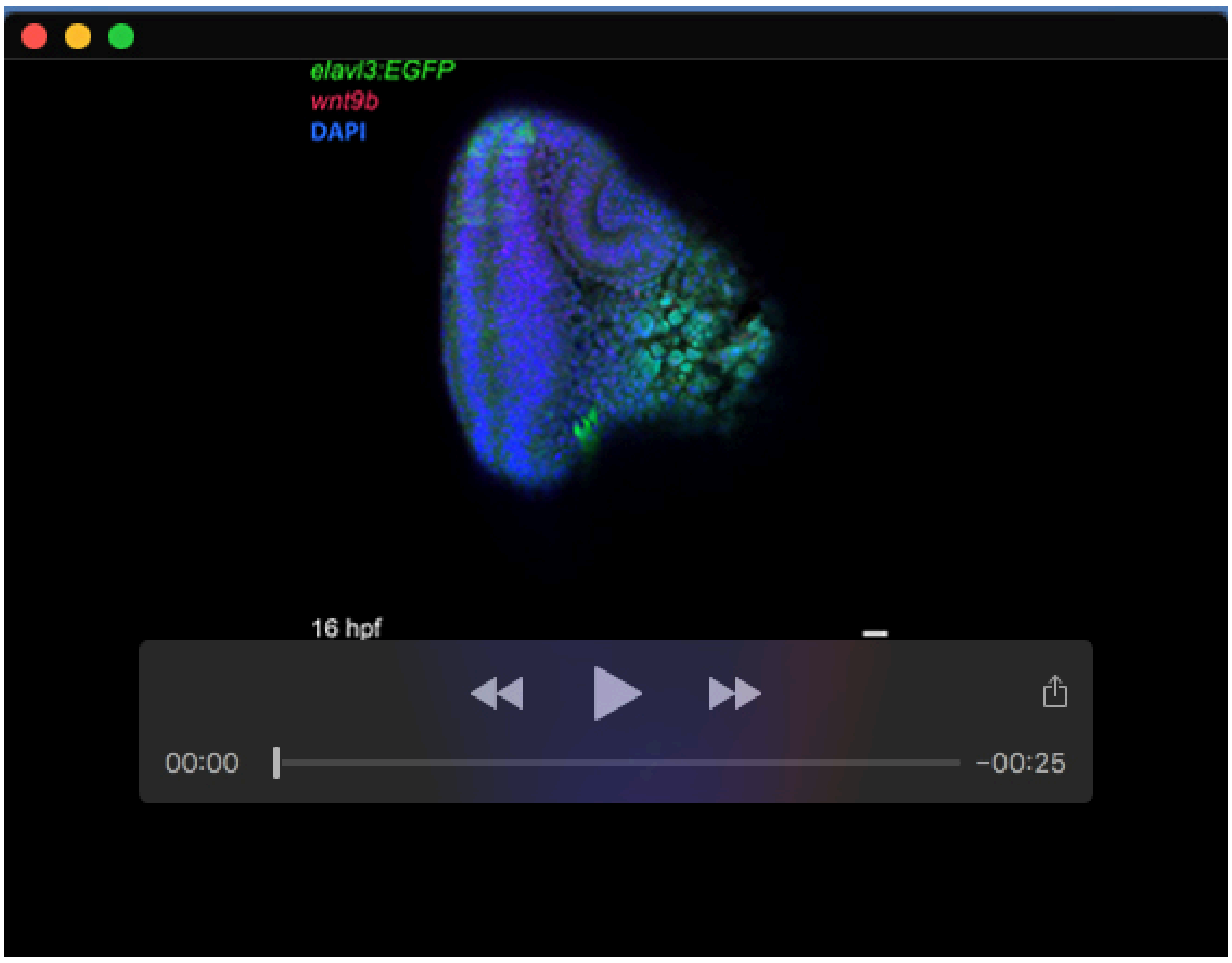

Movie 7. *wnt9b* is not co-expressed with the post-mitotic neuronal marker transgene Tg*(elavl3:EGFP)*^{knu3}, related to Fig. 2F,F'.

Scroll through a series of confocal z-planes from the dorsal towards ventral side of a 16 hpf embryo. Scale bar: 30 µm.

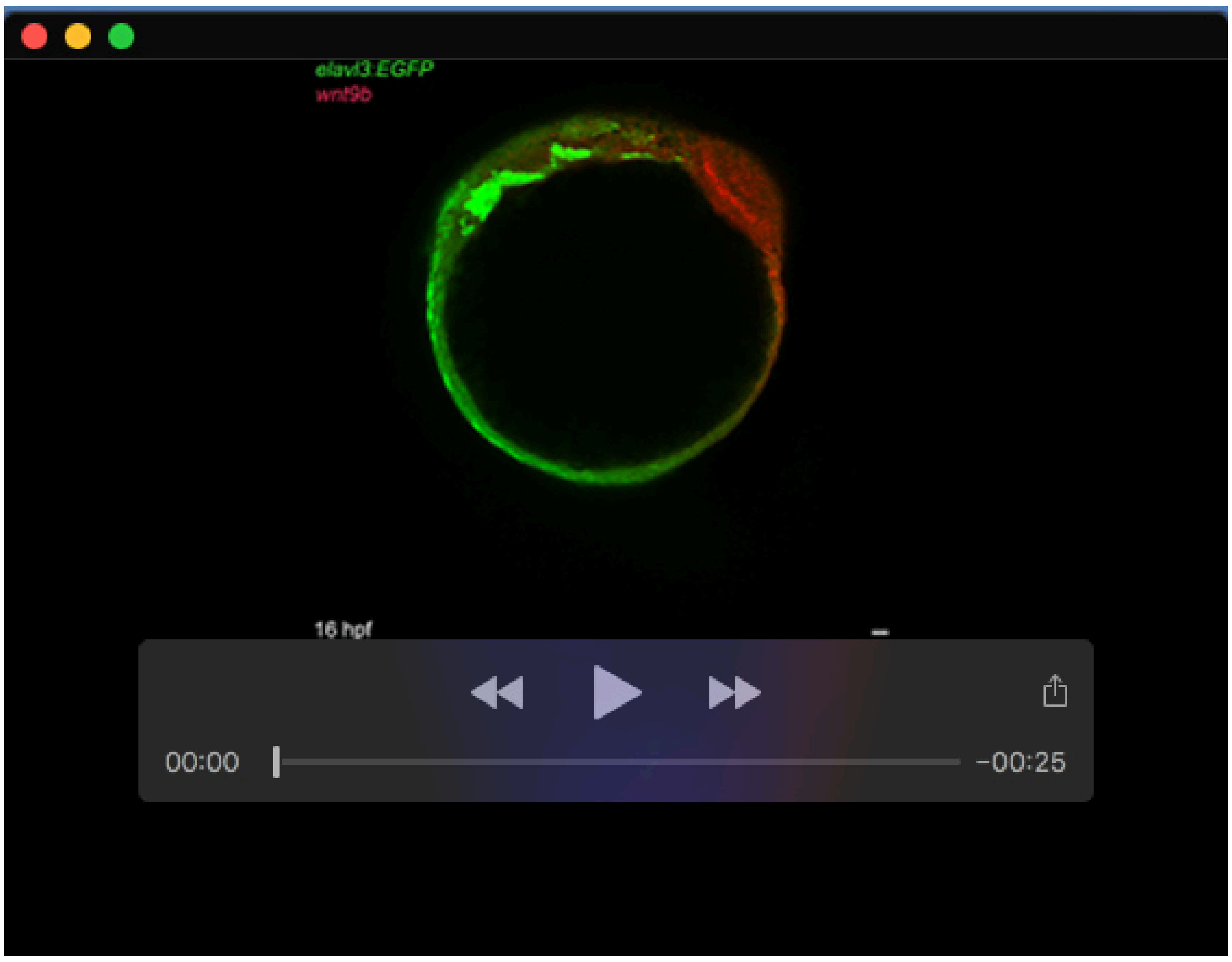

Movie 8. *wnt9b* is expressed in anterior and ventral regions of zebrafish embryos at 16 hpf, related to Fig. 2F,F'.

Scroll through a series of confocal z-planes from the dorsal towards ventral side of a 16 hpf embryo. The expression of the neuronal *Tg(elavl3:EGFP)*^{knu3} transgene is in posterior spinal cord neurons but not in anterior regions of embryos where *wnt9b* is expressed. Scale bar: 30 µm.

Development • Supplementary information
